# Randomized trial of activated vitamin D for acute kidney injury prevention in critically ill patients

**DOI:** 10.1172/jci.insight.193523

**Published:** 2025-09-09

**Authors:** David E. Leaf, Tushar Shenoy, Kevin Zinchuk, Shruti Gupta, Julie-Alexia Dias, Daniel Sanchez-Almanzar, Adit A. Ginde, Humra Athar, Changde Cheng, Tomoyoshi Tamura, Edy Y. Kim, Sushrut S. Waikar

**Affiliations:** 1 Division of Renal Medicine, Brigham and Women’s Hospital, Boston, Massachusetts, USA.; 2 Harvard Medical School, Boston, Massachusetts, USA.; 3 Investigational Drug Service, Brigham and Women’s Hospital, Boston, Massachusetts, USA.; 4 Department of Biostatistics, Harvard T.H. Chan School of Public Health, Boston, Massachusetts, USA.; 5 Department of Emergency Medicine, University of Colorado School of Medicine, Aurora, Colorado, USA.; 6 Division of Pulmonary and Critical Care Medicine, Brigham and Women’s Hospital, Boston, Massachusetts, USA.; 7 Division of Hematology and Oncology, Stem Cell Biology Program, Institute for Cancer Outcomes and Survivorship, Department of Biomedical Informatics and Data Science, University of Alabama at Birmingham, Birmingham, Alabama, USA.; 8 Department of Emergency and Critical Care Medicine, Keio University School of Medicine, Tokyo, Japan.; 9 Section of Nephrology, Boston Medical Center and Boston University Chobanian & Avedisian School of Medicine, Boston, Massachusetts, USA.

**Keywords:** Endocrinology, Immunology, Nephrology, Clinical trials, Monocytes, Transcriptomics

## Abstract

**BACKGROUND:**

Active vitamin D metabolites, including 25-hydroxyvitamin D (25D) and 1,25-dihydroxyvitamin D (1,25D), have potent immunomodulatory effects that attenuate acute kidney injury (AKI) in animal models.

**METHODS:**

We conducted a phase 2, randomized, double-blind, multiple-dose, 3-arm clinical trial comparing oral calcifediol (25D), calcitriol (1,25D), and placebo among 150 critically ill adult patients at high risk of moderate to severe acute kidney injury (AKI). The primary endpoint was a hierarchical composite of death, kidney replacement therapy (KRT), and kidney injury (baseline-adjusted mean change in serum creatinine), each assessed within 7 days following enrollment using a rank-based procedure. Secondary endpoints included new or progressive AKI and a composite of KRT or death. Hypercalcemia was the key safety endpoint. We also performed RNA-Seq on circulating CD14^+^ monocytes collected immediately prior to randomization and 2 days later.

**RESULTS:**

The global rank score for the primary endpoint was similar among calcifediol- (*n* = 51) versus placebo- (*n* = 49) treated patients (*P* = 0.85) and for calcitriol (*n* = 50) versus placebo-treated patients (*P* = 0.58). Secondary endpoints also occurred at similar rates across groups. Hypercalcemia occurred in 1 patient in the calcifediol group (1.7%), 1 patient in the calcitriol group (2.0%), and no patients in the placebo group. Compared with placebo, calcitriol upregulated more individual genes and pathways in circulating monocytes than did calcifediol, including pathways involving IFN-α, IFN-γ, oxidative phosphorylation, DNA repair, and heme metabolism.

**CONCLUSION:**

Treatment with calcifediol or calcitriol in critically ill adults upregulated multiple genes and pathways involving immunomodulation, DNA repair, and heme metabolism, but it did not attenuate AKI.

**TRIAL REGISTRATION:**

ClinicalTrials.gov (NCT02962102)

**FUNDING:**

NIH/NIDDK grant K23DK106448 (to DEL) and NIH/NHLBI grant R01HL16687 (to EYK).

## Abstract 2

In a randomized trial, treatment with activated vitamin D upregulated multiple genes and pathways but did not prevent acute kidney injury in critically ill patients.

## Introduction

**Figure 1:**
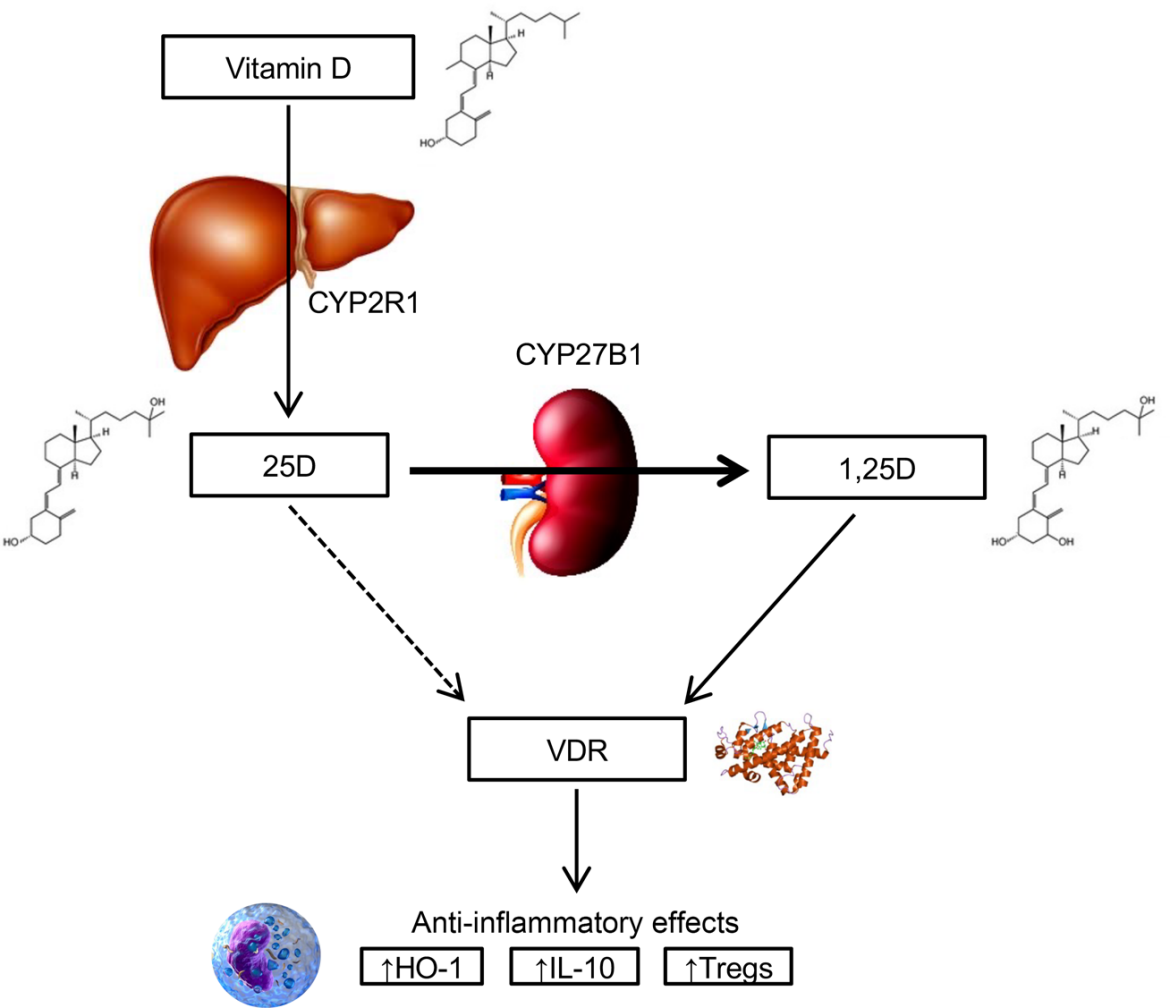
Overview of vitamin D metabolism. Vitamin D is derived from dietary sources and sunlight-induced cutaneous synthesis, and is converted to 25D in the liver. 25D, a partially activated vitamin D metabolite, is converted to its fully activated form, 1,25D, in the kidneys, immune cells, and other tissues by the cytochrome P450 enzyme, CYP27B1 (1-α hydroxylase). 1,25D binds to the cytoplasmic VDR, which is expressed nearly ubiquitously. The 1,25D-VDR complex translocates into the nucleus, where it binds to DNA sequence elements in vitamin D–responsive genes, ultimately influencing the expression of over 200 target genes. These include genes that encode antiinflammatory proteins, such as HO-1 and IL-10, as well as genes that upregulate production of Tregs, each of which attenuates acute organ injury in animal models. 25D can also bind to the VDR (dashed arrow). Its affinity for the VDR is several hundred–fold lower than that of 1,25D, but 25D circulates at ~1,000-fold higher concentrations than 1,25D. 1,25D, 1,25-dihydroxyvitamin D; 25D, 25-hydroxyvitamin D; HO-1, heme oxygenase-1; VDR, vitamin D receptor.

Vitamin D and its active metabolites play key roles in maintaining calcium and phosphate homeostasis. Beyond these “classical” effects, vitamin D metabolites also exert a variety of nonclassical actions, including potent immunomodulatory effects. These effects have been demonstrated in vitro, in animal models, and in humans, primarily with administration of the partially and fully activated vitamin D metabolites, 25-hydroxyvitamin D (25D) and 1,25-dihydroxyvitamin D (1,25D), respectively (Figure 1). 25D and 1,25D bind to the intracellular cytoplasmic vitamin D receptor (VDR), which is expressed nearly ubiquitously (1). This complex translocates into the nucleus, where it binds to DNA sequence elements in vitamin D–responsive genes, influencing the expression of over 200 target genes (2), including those affecting immunomodulatory pathways (3).

The potent immunomodulatory actions of vitamin D’s active metabolites have been leveraged in preclinical studies examining various acute organ injuries, including acute kidney injury (AKI). In animal models, prophylactic administration of 1,25D attenuates AKI induced by ischemia (4, 5), sepsis (6), and a variety of nephrotoxins (7–9). While the mechanisms are not fully understood, 1,25D has key antiinflammatory effects, including upregulation of heme oxygenase-1 (HO-1), IL-10, and Tregs (10–17), each of which are nephroprotective (18-24), along with downregulation of IL-6, TNF-α, and other proinflammatory cytokines (25, 26). Other implicated pathways for the nephroprotective effect of 1,25D include downregulation of the renin-angiotensin system, suppression of TGF-β signaling, and inhibition of NF-κB activation (27–30).

Attempts to translate these preclinical findings to humans have been scarce. Two randomized clinical trials examined the effects of large doses of vitamin D administered to critically ill patients and failed to demonstrate a reduction in hospital length of stay (31) or mortality (32); however, these studies used the precursor, vitamin D, rather than its active metabolites, 25D and 1,25D. Efficient conversion of vitamin D to 25D and 1,25D is dependent on intact liver and kidney function, respectively (Figure 1), organs that are often impaired in critical illness. Conversion of vitamin D to its active metabolites is further inhibited by the osteocyte-derived hormone, FGF23, which we and others reported is elevated in critically ill patients (33–36). Despite the compelling biologic rationale, few studies have administered 25D or 1,25D to critically ill patients as a therapeutic strategy to mitigate acute organ injury (37).

Accordingly, we conducted the Activated Vitamin D for the Prevention and Treatment of Acute Kidney Injury (ACTIVATE-AKI) trial. We hypothesized that treatment with activated vitamin D metabolites, calcifediol (25D) and calcitriol (1,25D), would reduce the incidence and severity of AKI among critically ill patients. We further hypothesized that calcifediol and calcitriol administration would affect immunomodulatory pathways, as they do in vitro, assessed by the transcriptomic profile of circulating monocytes.

## Results

### Clinical findings

#### Overview.

**Figure 2:**
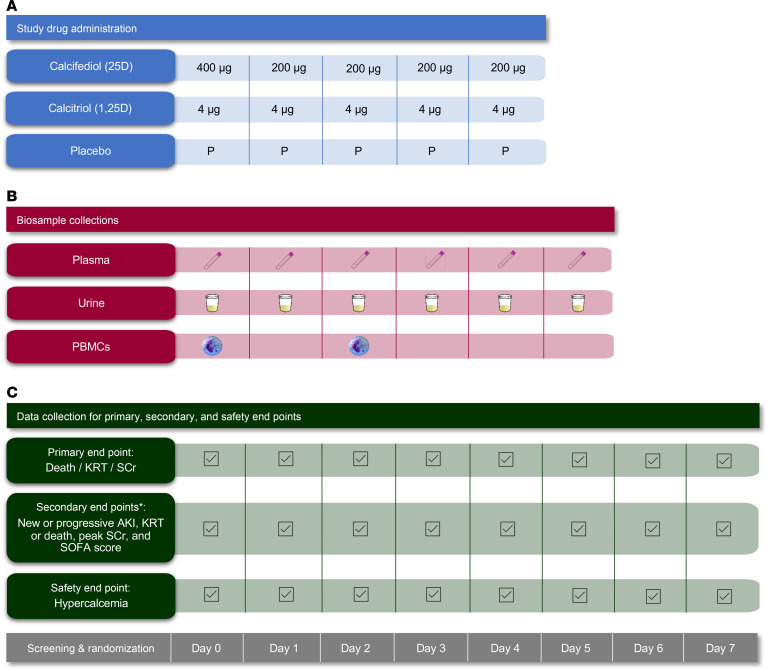
Trial schematic. (**A**) The study drug administrations on days 0 to 4. (**B**) The biosample collections on days 0 to 5. (**C**) Data collection for the primary, secondary, and safety endpoints on days 0 to 7. Asterisk indicates additional secondary endpoints included ICU and hospital LOS and 28-day all-cause mortality. AKI acute kidney injury; KRT, kidney replacement therapy; LOS, length of stay; PBMCs, peripheral blood mononuclear cells; SCr, serum creatinine; SOFA, Sequential Organ Failure Assessment.

We conducted a phase 2, randomized, double-blind, multiple-dose, parallel-group, 3-arm clinical trial comparing calcifediol (25D), calcitriol (1,25D), and placebo among critically ill adult patients admitted to intensive care units (ICUs) at Brigham and Women’s Hospital (BWH). Patients were eligible if they were at high risk of developing moderate-to-severe AKI within 7 days, based on either the presence of mild AKI (Kidney Disease: Improving Global Outcomes [KDIGO] stage 1) (38) or an elevated AKI risk score (Supplemental Table 1; supplemental material available online with this article; https://doi.org/10.1172/jci.insight.193523DS1). The full list of eligibility criteria is provided in Supplemental Table 2. Patients were randomly assigned (1:1:1) to receive oral/enteral calcifediol, calcitriol, or placebo once daily for 5 days (Figure 2A). Biosamples were collected on enrollment (day 0, prior to receipt of study drug) and on a daily basis for the next 5 days (Figure 2B). Data for the primary endpoint, key secondary endpoints, and safety endpoints were assessed through day 7 (Figure 2C).

#### Patients.

**Figure 3:**
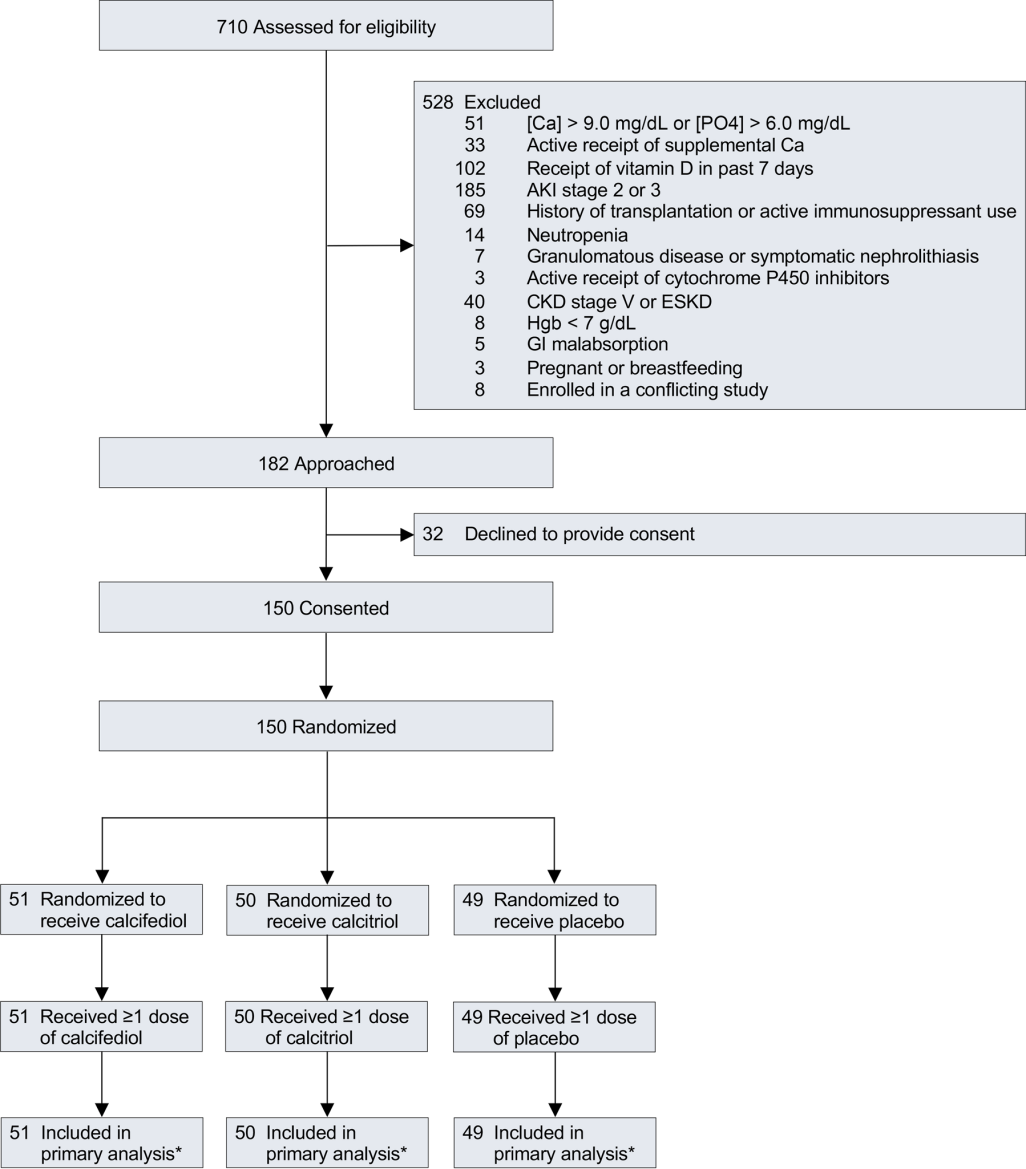
Screening, enrollment, and randomization. AKI, acute kidney injury; Ca, calcium; CKD, chronic kidney disease; ESKD, end-stage kidney disease; GI, gastrointestinal; Hgb, hemoglobin; PO4, phosphate. Asterisk indicates that no patients were lost to follow-up.

From April 2017 through July 2020, we screened 710 patients, approached 182 who met eligibility criteria, and obtained consent from 150 (Figure 3). In total, 51 patients were assigned to the calcifediol group, 50 to the calcitriol group, and 49 to the placebo group (Figure 3). Baseline characteristics were similar among the 3 groups (Tables 1 and 2 and Supplemental Table 3). The most common reasons for ICU admission were respiratory failure, hypotension/shock, and sepsis. Most patients were enrolled within 2 days of hospital admission and within 1 day of ICU admission (Tables 1 and 2). In all 3 groups, over 80% of patients were receiving invasive mechanical ventilation, over 50% were receiving vasopressors/inotropes, and approximately 35% had AKI on enrollment (Tables 1 and 2). All patients received at least 1 dose of the study drug to which they were initially assigned (Supplemental Figure 1).

#### Plasma vitamin D metabolite levels.

**Figure 4:**
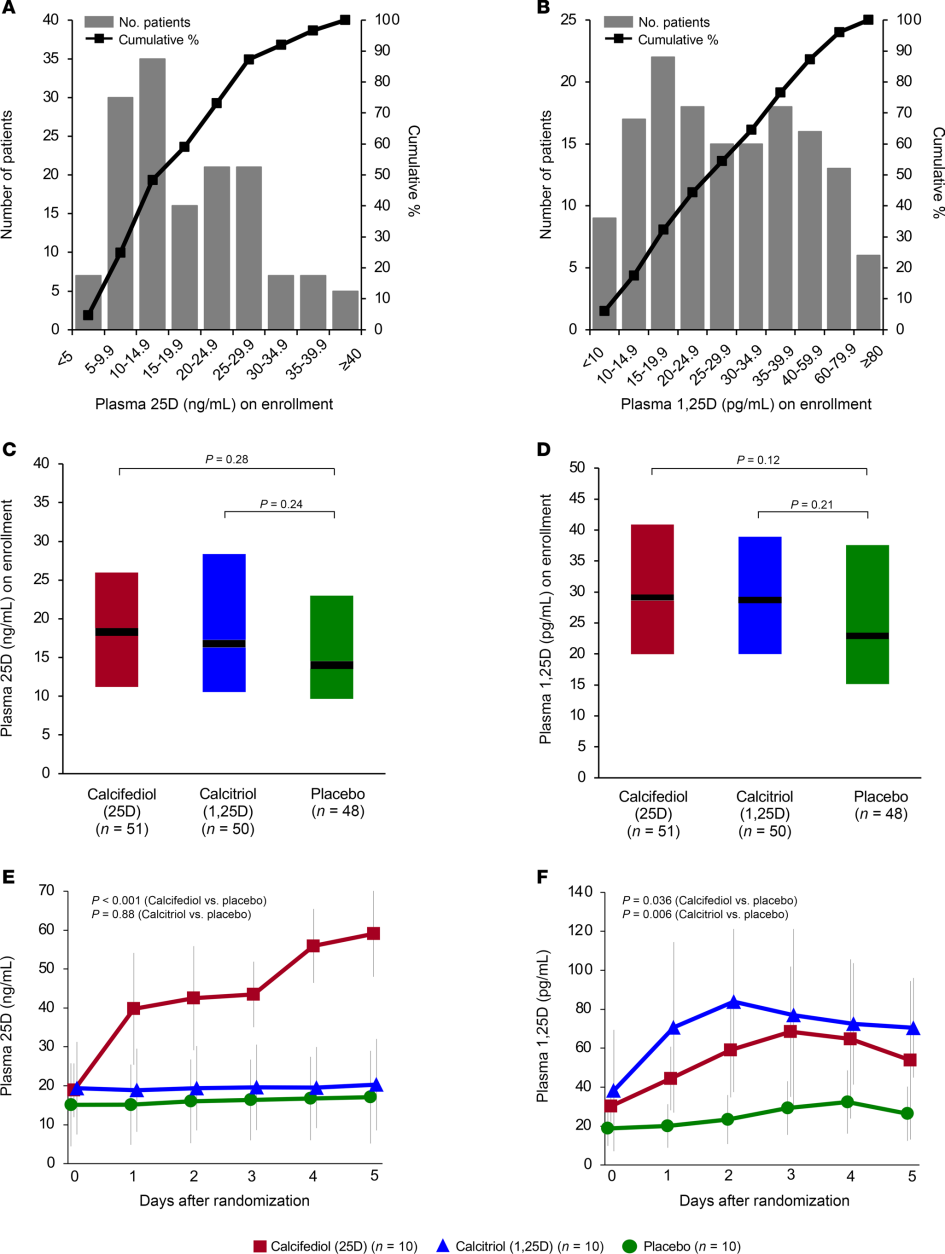
Vitamin D metabolite levels on enrollment and follow-up. (**A** and **B**) The distribution of plasma 25D and 1,25D levels in all study patients on enrollment, respectively. (**C** and **D**) Median (IQR) plasma levels of 25D and 1,25D on enrollment, respectively, according to treatment group. (**E** and **F**) Median (IQR) plasma levels of 25D and 1,25D, respectively, assessed longitudinally in a random subcohort. *P* values in **C** and **D** are derived from the Wilcoxon rank-sum test, and those in **E** and **F** are derived from mixed linear models for repeated measures. 1,25D, 1,25-dihydroxyvitamin D; 25D, 25-hydroxyvitamin D; IQR, interquartile range.

The distribution of plasma 25D levels on enrollment is shown in Figure 4A. Of the 149 patients with 25D levels measured on enrollment, 130 (87.2%) were vitamin D insufficient (25D < 30 ng/mL), 88 (59.1%) were vitamin D deficient (25D < 20 ng/mL), and 37 (24.8%) were severely vitamin D deficient (25D < 10 ng/mL) (Figure 4A). The distribution of plasma 1,25D levels on enrollment is shown in Figure 4B. Of the 149 patients with 1,25D levels measured on enrollment, 66 (44.3%) had a level < 20 pg/mL, the lower end of the reference range (20–60 pg/mL) (Figure 4B). Median plasma 25D and 1,25D levels at enrollment were 16.1 ng/mL (interquartile range [IQR], 10.2–25.9) and 26.9 pg/mL (IQR, 17.5–39.1), respectively, and were similar among treatment groups (Figure 4, C and D).

#### Longitudinal plasma vitamin D metabolite levels.

In the subcohort of patients who had vitamin D metabolite levels measured longitudinally, plasma 25D levels increased 2-fold by day 1 and 3-fold by day 5 among calcifediol-treated patients, but they were unchanged in the calcitriol- and placebo-treated patients (Figure 4E). Plasma 1,25D levels increased more than 2-fold by day 2 among calcitriol-treated patients, increased to a lesser extent among calcifediol-treated patients, and were relatively unchanged among placebo-treated patients (Figure 4F).

#### Primary endpoint.

Overall, the primary endpoint — a hierarchical composite of death, kidney replacement therapy (KRT), and baseline-adjusted mean change in serum creatinine (SCr) within 7 days — was no different among groups (*P* = 0.85, calcifediol versus placebo; *P* = 0.58, calcitriol versus placebo; Table 3). Death within 7 days occurred in 4 patients (7.8%) in the calcifediol group, 9 (18.0%) in the calcitriol group, and 6 (12.2%) in the placebo group. AKI requiring KRT within 7 days occurred in 1 patient (2.0%) in the calcifediol group, 1 (2.0%) in the calcitriol group, and 4 (8.2%) in the placebo group. The median baseline-adjusted mean changes in SCr in the 7 days following randomization were close to zero in all 3 groups, with IQRs spanning from –21.5% to 18.3%, indicating that most patients did not experience clinically relevant new or worsening AKI following enrollment (Table 3).

Subgroup analyses for the primary endpoint are shown in Supplemental Table 4. There was effect modification according to ICU type, whereby the calcitriol group had more evidence of kidney injury (higher global rank score) compared with placebo among those admitted to medical ICUs (*P* = 0.012) and less evidence of kidney injury compared with placebo among those admitted to surgical ICUs (*P* = 0.014). No other between-group differences were noted.

#### Secondary endpoints.

Secondary endpoints are shown in Table 3. Calcifediol-treated patients had a numerically lower rate of KRT or death within 7 days compared with placebo, and those in both the calcifediol- and calcitriol-treated groups had trends toward a shorter ICU length of stay, but none of these differences reached statistical significance (Table 3). All other secondary endpoints were similar among groups (Table 3), as was the longitudinal Sequential Organ Failure Assessment (SOFA) score (Supplemental Figure 2).

#### Safety.

Hypercalcemia occurred in 1 patient in the calcifediol group (1.7%) and in 1 patient in the calcitriol group (2.0%) (Supplemental Figure 3A). Serum calcium levels peaked at 11.6 mg/dL on day 7 in the calcifediol-treated patient and at 12.6 mg/dL on day 5 in the calcitriol-treated patient. Hyperphosphatemia occurred in 3 patients in the calcifediol group (5.9%), 9 in the calcitriol group (18.0%), and 1 in the placebo group (2.0%) (Supplemental Figure 3B). When assessed longitudinally, serum calcium and phosphate levels were highest in calcitriol-treated patients (Supplemental Figure 3, C and D).

#### Markers of kidney injury.

Longitudinal plasma and urinary Kidney Injury Molecule-1 (KIM-1) levels were generally similar among groups (Supplemental Figure 4, A and B), though plasma KIM-1 was marginally higher among calcifediol- versus placebo-treated patients (*P* = 0.052).

### Transcriptomic analysis of monocytes

#### Overview.

**Figure 5:**
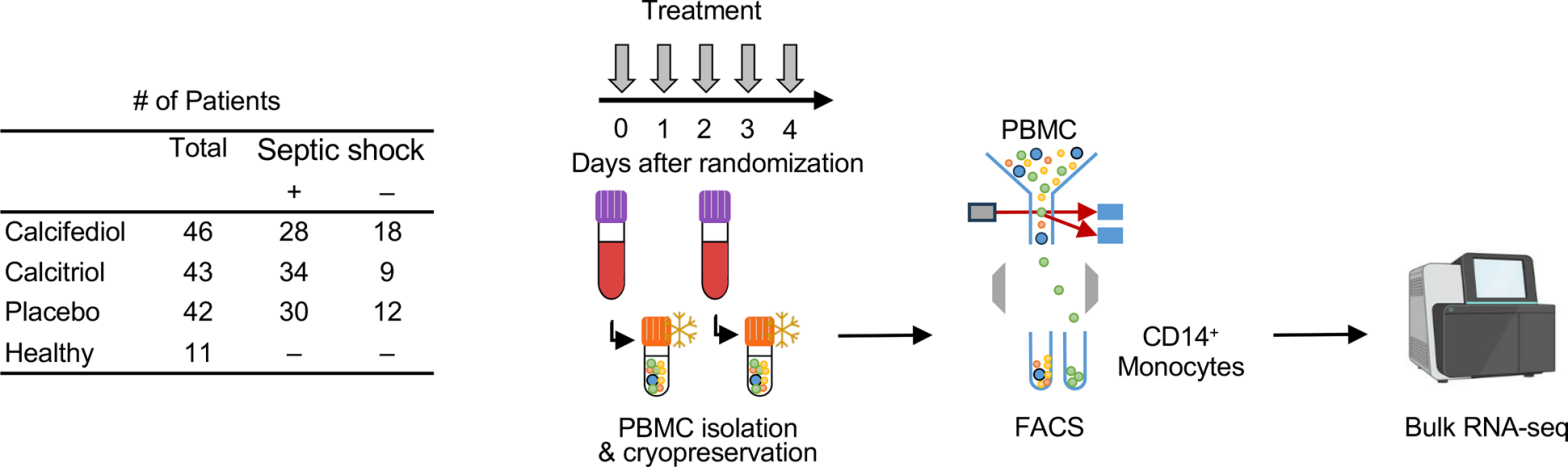
Overall experimental design for transcriptomic analysis of monocytes. Peripheral blood mononuclear cells (PBMCs) were isolated from patients on day 0 (prior to treatment) and day 2 after randomization. CD14^+^ monocytes were isolated by flow cytometry sorting and analyzed by bulk RNA-Seq.

We next examined changes in gene expression of circulating monocytes in response to calcifediol and calcitriol treatment in vivo, comparing fold-change in gene expression on day 0 to day 2 for each patient. We focused on monocytes, as they express the cellular machinery (such as the vitamin D receptor and CYP27B1) required to respond to calcifediol and calcitriol (39–42). We first used flow cytometry to sort CD14^+^ monocytes from peripheral blood mononuclear cells (PBMCs) collected on days 0 and 2 (Figure 5 and Supplemental Figure 5), followed by bulk RNA-Seq. Our final analysis consisted of 131 patients with paired PBMC samples from days 0 and 2, including 46 patients treated with calcifediol, 43 with calcitriol, and 42 with placebo. Transcriptional changes in monocytes were examined under 3 broad analyses: prespecified genes, unbiased global transcriptome, and gene set enrichment.

#### Prespecified genes.

**Figure 6:**
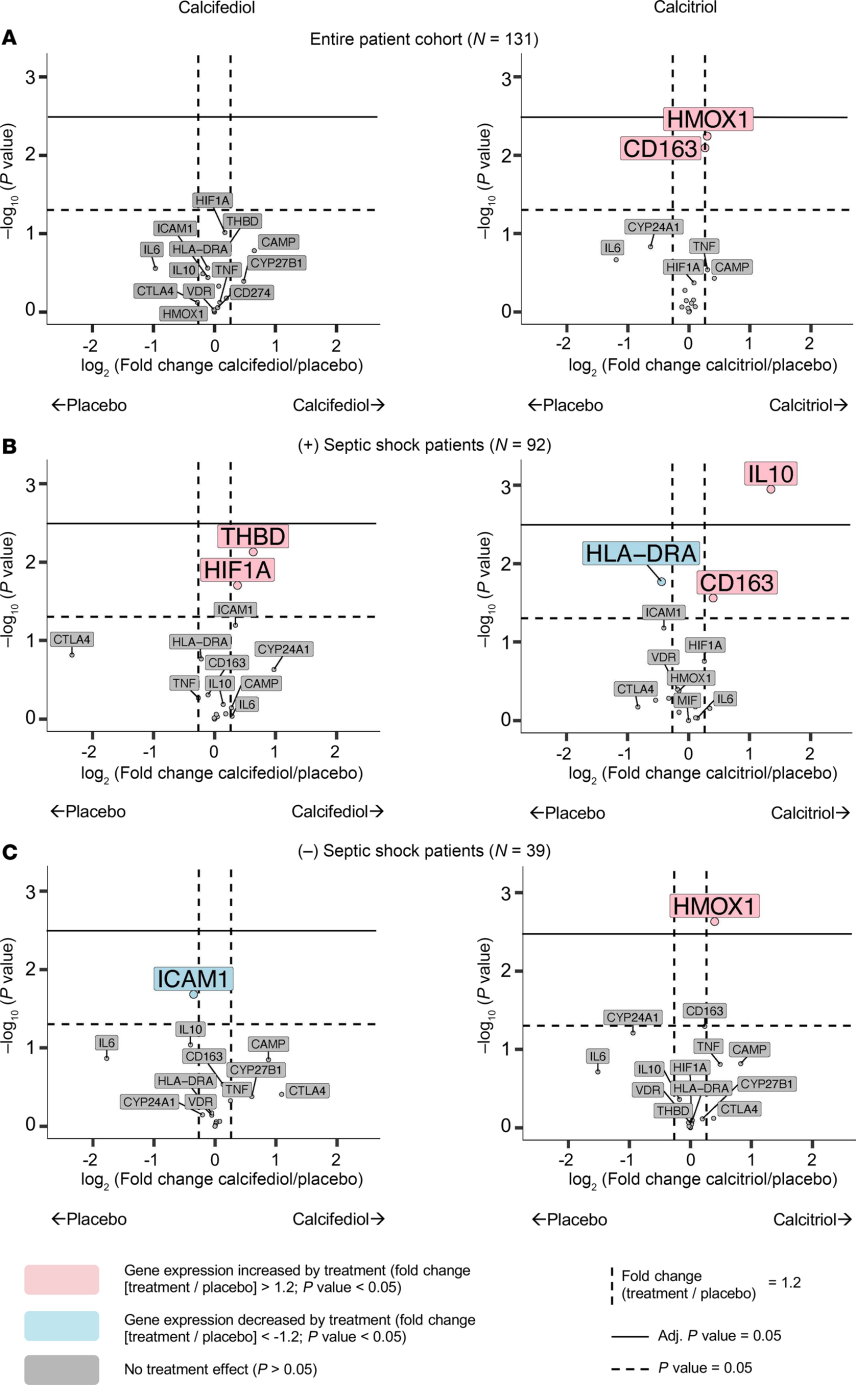
Effect of calcifediol and calcitriol on monocyte expression of prespecified genes in patients with versus without septic shock. (**A**–**C**) The treatment effect of calcifediol and calcitriol versus placebo on monocyte expression of 16 genes prespecified prior to initiation of the clinical trial. The fold-change of gene expression between day 0 (pretreatment) and day 2 (postinitiation of treatment) was calculated. The relative log_2_fold-change between treatment and placebo (*x* axis) and unadjusted and adjusted *P* values (*y* axis) are shown. Data are shown for the entire cohort (**A**), those with septic shock (**B**), and those without septic shock (**C**).

Neither calcifediol nor calcitriol had a significant effect on any of the 16 prespecified genes compared to placebo (Figure 6A and Supplemental Tables 5 and 6). Calcitriol resulted in a trend of 1.2-fold increased expression of both *HMOX1* and *CD163* (unadjusted *P* < 0.05, adjusted *P* > 0.05).

We hypothesized that treatment effects may have been obscured by the heterogeneity of the patient population. Therefore, in a post hoc analysis, we stratified our analyses according to septic shock, defined as sepsis with receipt of vasopressors, at study entry. Septic shock is associated with a ~40% in-hospital mortality, approximately 4 times higher than the mortality seen with sepsis alone (43). Septic shock was present on enrollment in 92 patients and absent in 39 (Figure 5). Stratifying patients by the presence of septic shock revealed distinct effects of calcitriol. In patients with septic shock, calcitriol increased the expression of *IL-10* (2.6-fold, adjusted *P* < 0.05) (Figure 6B), whereas in those without septic shock, calcitriol increased the expression of *HMOX1* (1.3-fold, adjusted *P* < 0.05) (Figure 6C and Supplemental Table 6).

#### Global differential expression of genes.

We next examined the global transcriptome of circulating monocytes in response to calcifediol and calcitriol, focusing on the 906 genes that were differentially expressed between patients on day 0 versus healthy controls. Treatment with calcifediol and calcitriol significantly affected the expression of multiple genes compared with placebo. These included genes that regulate inflammation (*NFIL3*, *GLDN*, *MSR1*), macrophage infiltration and M1 polarization (*CLU*), the complement pathway (*C1QA/B/C*), and IFN-response (*IFITM3*) (Supplemental Figure 6A). Stratifying patients according to septic shock on enrollment again revealed distinct effects of calcifediol and calcitriol (Supplemental Figure 6, B and C). A complete list of genes significantly regulated by calcifediol and calcitriol, both overall and stratified by septic shock, is provided in the Supporting Data Values file.

#### Gene set enrichment analysis (GSEA).

**Figure 7:**
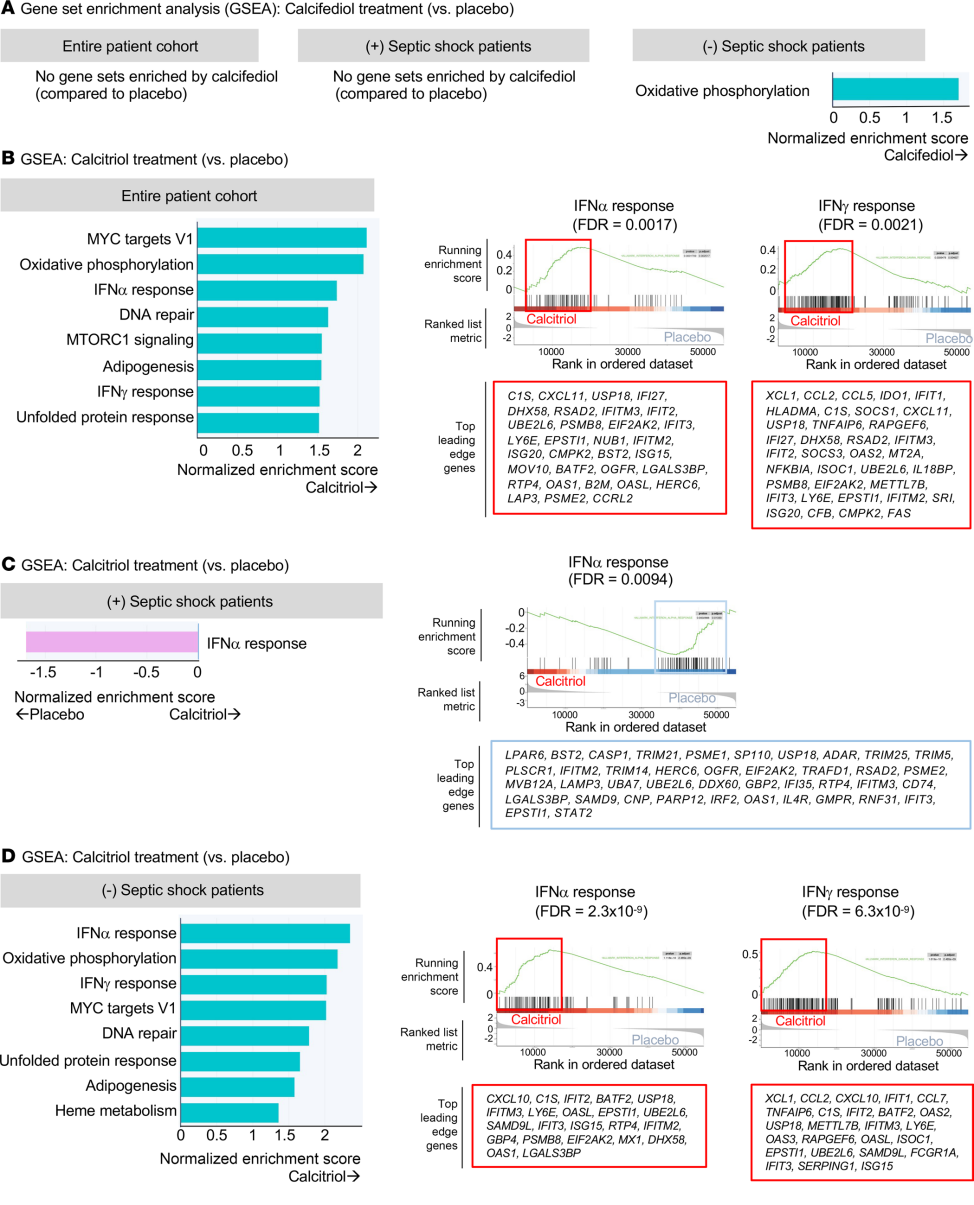
Effect of calcifediol and calcitriol treatment on monocyte gene set enrichment in patients with versus without septic shock. Peripheral blood mononuclear cells (PBMCs) were isolated from patients on day 0 (prior to treatment) and day 2 after randomization. CD14^+^ monocytes were isolated by flow cytometry sorting and analyzed by bulk RNA-Seq. Gene set enrichment analysis (GSEA) was performed for treatments versus placebo. (**A**) GSEA for calcifediol treatment versus placebo. (**B**–**D**) GSEA for calcitriol treatment versus placebo and leading edge genes for selected gene sets are shown for all patients (**B**), patients with septic shock (**C**), and patients without septic shock (**D**).

We next elucidated the pathways enriched by calcifediol or calcitriol treatment. Across the entire cohort, calcifediol treatment did not result in significant gene set enrichment compared with placebo (adjusted *P* < 0.05; Figure 7A). In contrast, calcitriol treatment enriched several gene sets, including inflammatory pathways (IFN-α and IFN-γ responses), metabolism (oxidative phosphorylation, MTORC1 signaling), and stress (DNA repair, unfolded protein response) (Figure 7B).

When stratified by the presence of septic shock, calcifediol treatment did not result in gene set enrichment in patients with septic shock (Figure 7A) but resulted in enrichment of the oxidative phosphorylation pathway in those without septic shock (Figure 7A). Calcitriol treatment, in contrast, resulted in reduced IFN-α response in patients with septic shock (Figure 7C), whereas those without septic shock drove most of the response to calcitriol seen in the entire cohort (Figure 7D). As seen in the entire patient cohort (Figure 7B), those without septic shock had increased expression of IFN-α, IFN-γ, oxidative phosphorylation, DNA repair, and unfolded protein response pathways in response to calcitriol (Figure 7D). In addition, they had increased expression of the heme metabolism pathway (Figure 7D), consistent with the upregulation of *HMOX1* seen in the analysis of prespecified genes (Figure 6C).

#### Exploratory analyses, stratified by sex.

Finally, we conducted an exploratory analysis to assess whether the monocyte transcriptomic response to calcifediol or calcitriol differed by sex. Transcriptional changes in monocytes according to sex are shown for prespecified genes (Supplemental Figures 7 and 8), unbiased global transcriptome (Supplemental Figure 9), and gene set enrichment (Supplemental Figure 10). Each of these analyses revealed distinct differential gene expression by sex.

## Discussion

In this 3-arm, randomized, double-blind, placebo-controlled trial, we found that administration of calcifediol and calcitriol increased circulating levels of 25D and 1,25D by up to 3-fold, respectively, but did not attenuate the incidence or severity of AKI among critically ill adults. Similarly, neither calcifediol nor calcitriol affected secondary clinical outcomes. By examining the transcriptome of circulating monocytes, we found that calcitriol influenced the expression of multiple genes and gene pathways — including those involved in inflammation, macrophage infiltration and M1 polarization, the complement pathway, IFN, oxidative phosphorylation, DNA repair, and heme metabolism — each of which play major roles in acute organ injury. These effects on gene expression were particularly pronounced among patients without septic shock. Cumulatively, these findings suggest that vitamin D metabolites exert specific immunomodulatory effects in distinct patient populations; however, these effects did not translate into a detectable clinical benefit in a heterogeneous population of critically ill patients in this phase 2 clinical trial.

In preclinical models, a therapeutic role of vitamin D and its metabolites on the attenuation of acute organ injury has been demonstrated across a wide range of species and models, including acute lung injury, sepsis, and AKI (4–9, 44–46). The vast majority of these studies examined the activated vitamin D metabolite, calcitriol (1,25D). However, the 2 largest clinical trials in critically ill patients used the precursor molecule, vitamin D, rather than its active metabolites, 25D and 1,25D (31, 32). Similarly, studies conducted in hospitalized patients at risk for severe illness from COVID-19 also used the precursor, vitamin D, and these studies likewise failed to show a benefit (47, 48). However, efficient conversion of vitamin D to 25D and 1,25D is dependent on intact liver and kidney function (Figure 1) and may be further inhibited by elevated circulating levels of FGF23 (33–36). Thus, these studies left open the possibility that administration of activated vitamin D metabolites could be beneficial.

We demonstrated that administration of calcifediol and calcitriol raises plasma 25D and 1,25D levels, respectively, considerably faster and to a greater extent than can be achieved with even very large doses of vitamin D precursor (e.g., > 500,000 IU). Despite these favorable pharmacokinetic properties, few studies of activated vitamin D metabolites have been performed in critically ill patients. Those that have been conducted have been limited by small sample size and short dosing regimens, often with a single dose of activated vitamin D administered, despite calcitriol having a half-life of only 5–10 hours. For example, we previously randomized 67 critically ill patients with severe sepsis or septic shock to receive a single i.v. dose of calcitriol (2 μg) or placebo. We found that 24 hours after study drug administration, calcitriol-treated patients had higher leukocyte mRNA expression of the antimicrobial protein, cathelicidin, as well as IL-10, compared with placebo (49). However, the study only assessed a single dose of calcitriol and was underpowered to evaluate clinical outcomes.

The current study expands on prior findings in several important ways. First, we simultaneously examined 2 forms of activated vitamin D — calcifediol and calcitriol — bypassing the requirement for hepatic and renal conversion, resulting in rapid and large increases in circulating 25D and 1,25D levels. Second, we examined repeated dosing on a daily basis for 5 days, ensuring a sustained increase in circulating 25D and 1,25D levels. Third, we included patients both at risk of developing AKI as well as those who already had early AKI, reflecting real-world clinical practice where interventions often cannot be administered strictly on a prophylactic basis. Fourth, we used a hierarchical composite outcome to account for the competing risks of death and KRT in assessing kidney injury. Finally, we included a comprehensive translational component to our study by examining the effect of calcifediol and calcitriol on the transcriptome of circulating monocytes.

Immunomodulatory effects of activated vitamin D metabolites have been demonstrated in vitro and in various populations (49–53), but the current study is the first, to our knowledge, to perform RNA-Seq on circulating monocytes in critically ill patients — a highly vulnerable population for whom development of novel therapies is urgently needed. We found that treatment with calcifediol and calcitriol modified the transcriptome of monocytes in vivo, as they do in vitro. Both treatments increased the expression of several immunomodulatory genes implicated in the responses to a range of diseases. Compared with calcifediol treatment, calcitriol upregulated more individual genes and pathways. Patient heterogeneity revealed distinct treatment effects among subcohorts, with different effects observed in those with versus without septic shock. In patients with septic shock, calcitriol increased the expression of *IL-10* and reduced IFN-α response. In contrast, in those without septic shock calcitriol upregulated several pathways, including IFN-α, IFN-γ, oxidative phosphorylation, DNA repair, and heme metabolism. These findings raise the possibility that a precision-medicine approach — using the right drug at the right dose in the right population — would have been more likely to result in therapeutic benefit. Future studies are needed to explore such a possibility.

We acknowledge several limitations. First, this was a single-center study, and the sample size was modest. Accordingly, we were underpowered to detect effects on secondary endpoints, such as the composite of KRT or death, for which there was a trend toward benefit in the calcifediol group. Similarly, we were underpowered to examine the interaction between clinical features (e.g., sex, septic shock) and the monocyte transcriptomic response to calcifediol or calcitriol, though our preliminary analyses revealed distinct responses in each of these groups. Second, despite the clinical enrichment used for kidney injury, relatively few patients experienced a clinically relevant increase in SCr during the initial 7 days following randomization. Third, we recruited a heterogeneous population of critically ill patients, including those from both medical and surgical ICUs, those with and without sepsis and septic shock, and those with and without early evidence of AKI on enrollment. As noted above, it is possible that a therapeutic benefit would have been more likely to be observed in a more homogeneous population. Fourth, clinical endpoints focused on renal outcomes, ICU and hospital length of stay, and mortality. Given the important in vitro effects of vitamin D metabolites on immune defense, future studies should assess endpoints related to immunological response and infectious complications. Finally, we assessed monocyte transcriptomics at 2 time points: on enrollment and 2 days later. Given the dynamic nature of the transcriptome, future studies should examine both earlier and later time points following administration of immunomodulatory agents such as activated vitamin D metabolites.

In summary, we found that administration of the activated vitamin D metabolites, calcifediol and calcitriol, resulted in a rapid and sustained increase in circulating levels of 25D and 1,25D, respectively, but did not attenuate the incidence or severity of AKI among critically ill adults. Calcitriol affected the expression of multiple genes and gene pathways relevant to acute organ injury, and these effects were observed to a much greater extent in patients without septic shock. Future studies should consider examining the immunomodulatory effects of calcitriol in a more homogeneous population (e.g., those without septic shock) to further explore its therapeutic potential in attenuating acute organ injury.

## Methods

### Sex as a biological variable

Both male and female sexes were included. The study design accounted for sex as a biological variable through subgroup analyses of the primary endpoint stratified according to sex.

### Trial design and oversight

We conducted a phase 2, randomized, double-blind, multiple-dose, parallel-group, 3-arm clinical trial comparing calcifediol (25D), calcitriol (1,25D), and placebo among critically ill adult patients admitted to ICUs at BWH. Patients were randomly assigned (1:1:1) to receive oral/enteral calcifediol, calcitriol, or placebo once daily for 5 days. Randomization was performed using permuted blocks of 3 and stratified by the presence of AKI on enrollment. AKI was defined according to the KDIGO Work Group criteria (38) as an increase in SCr ≥ 0.3 mg/dL in the previous 48 hours (h), ≥ 50% in the previous 7 days, or urine output (UOP) < 0.5 mL/kg/h for 6 consecutive hours during the previous 24h. Trial personnel, clinical staff, and patients were blinded to treatment group assignment.

Calcifediol active product ingredient was supplied as a gift by CARBOGEN AMCIS B.V. (previously Dishman Netherlands B.V., Veenendaal, the Netherlands). Calcifediol crystals were levigated and dissolved in a small volume of propylene glycol USP (Letco) and then diluted in medium chain triglyceride (MCT) oil (Nestle Health Science), with a final concentration of 100 μg/mL. To confirm potency, purity, and 6-month stability, we developed and validated a Master Formulation Record (MFR) to compound calcifediol solution locally in the Investigational Drug Service at BWH. Method validation was performed using a high-performance liquid chromatography-based assay (DynaLabs) to quantify 25D levels over time and assess for the accumulation of unwanted bioproducts of calcifediol degradation. The finalized MFR and the results of the associated method validation were submitted to the Food and Drug Administration (FDA) as part of an Investigational New Drug (IND) application (no. 133057). Calcitriol is commercially available and was used without modification (Roxane Laboratories, 1 μg/mL).

### Patients

Eligible patients were: (a) 18 years of age or older; (b) admitted to an ICU within 48 hours prior to enrollment; (c) likely to remain in the ICU for ≥ 72 hours according to the judgement of the clinical team; (d) were able to swallow, or had a naso/orogastric or gastrostomy tube in place; and (e) were at high risk of developing moderate-to-severe AKI within 7 days. The latter was defined as either the presence of mild AKI (KDIGO stage 1) (38) or an AKI risk score ≥ 6 points, utilizing a modified prediction score (54) that incorporates both acute and chronic risk factors (Supplemental Table 1).

Key exclusion criteria were: (a) total serum calcium > 9.0 mg/dL; (b) serum phosphate > 6.0 mg/dL; (c) active receipt of oral calcium; (d) receipt of vitamin D > 1,000 IU/day, or 25D or 1,25D (at any dose) in the previous 7 days; (e) KDIGO AKI stages 2 or 3 (defined as a relative increase in SCr ≥ 100% in the previous 7 days, UOP < 0.5 mL/kg/h for ≥ 12h, or receipt of KRT); (f) chronic kidney disease stage 5 (baseline estimated glomerular filtration rate [eGFR] < 15 mL/min/1.73m^2^) or end-stage kidney disease; and (g) gastrointestinal malabsorption. The full list of exclusion criteria is provided in Supplemental Table 2.

### Procedures

Calcifediol was administered as an oral liquid solution of 400 μg (in 4 mL) on day 0 (the day of enrollment) and 200 μg (in 2 mL) on days 1 through 4. Calcitriol was administered as an oral liquid solution of 4 μg (in 4 mL) daily on days 0 through 4 (Figure 2A). Placebo consisted of MCT oil and had an appearance, consistency, and smell similar to the active treatments. Because the volume of calcifediol differed from that of calcitriol on days 1 through 4, a double-dummy design (4 mL + 2 mL) was used to maintain blinding. Dosing regimens were designed with consideration of efficacy, safety, and pharmacokinetics (i.e., the expected circulating levels of 25D and 1,25D that would be achieved). Additional data supporting the dosing regimens used are provided in the Supplemental Data 1.

### Endpoints and assessments

#### Primary endpoint.

The primary endpoint was a hierarchical composite of death, KRT, and kidney injury (baseline-adjusted mean change in SCr), each assessed within 7 days following enrollment using a rank-based procedure. The highest rank was assigned to those who died. The second highest rank was assigned to those who survived and received KRT. Those who survived and did not receive KRT were ranked according to their baseline-adjusted average change in SCr. The rationale for this endpoint was to: (a) prioritize each patient’s most clinically relevant outcome for inclusion in the analysis (55); (b) account for KRT and death as competing risks for AKI (56); and (c) assess average SCr levels, rather than simply their peak, to account not only for each patient’s maximum AKI severity but also its duration (57). A similar endpoint has been used in previous AKI clinical trials (58–60).

#### Subgroup analyses.

We examined the primary endpoint in the following 7 subgroups: age (< 65 versus ≥ 65 years); sex; AKI on enrollment (present versus absent); ICU type (medical versus surgical); APACHE II score (61) (above versus below the median value on enrollment); septic shock on enrollment (present versus absent); and 25D level (above versus below the median value on enrollment).

#### Secondary endpoints.

Secondary endpoints included: (a) individual components of the primary endpoint; (b) new or progressive AKI within 7 days; (c) KRT or death within 7 days; (d) peak SCr within 7 days; (e) ICU- and hospital- length of stay; (f) longitudinal SOFA score (62), assessed on the day of enrollment and daily for the next 7 days (details on SOFA score calculation are provided in the Supplemental Methods); and (g) 28-day all-cause all-location mortality. To account for death as a competing risk, ICU and hospital length of stay were assessed both as absolute values and as ICU- and hospital-free days. The latter was calculated as 28 minus the number of ICU or hospitalization days, respectively, assuming survival to 28 days. Patients who died before 28 days were assigned a score of zero (63).

#### Safety.

The primary safety endpoint was hypercalcemia, defined as serum calcium > 10.7 mg/dL in the first 7 days following randomization. We also assessed hyperphosphatemia, defined as serum phosphate > 6.0 mg/dL in the first 7 days following randomization. Finally, we assessed serum calcium and phosphate levels longitudinally in the first 7 days. Throughout the trial, an independent data and safety monitoring board reviewed safety data and recommended continuation of the trial to completion.

#### Biosample collections and measurement of vitamin D metabolites and markers of kidney injury.

EDTA-plasma and urine were collected on enrollment (day 0, prior to receipt of study drug) and on a daily basis for the next 5 days (Figure 2B). Samples were centrifuged and stored at –80°C. Plasma 25D and 1,25D levels were measured at the University of Washington Analytic Core using immunoaffinity enrichment and liquid chromatography–tandem mass spectrometry (64). 25D and 1,25D levels were measured on day 0 in all 150 patients, and at each of the 6 sample collection time points in 30 randomly selected patients (10 from each treatment group). Plasma and urinary KIM-1 levels were measured in all 150 patients on days 0, 1, 2, and 3. Urinary KIM-1 levels were normalized to urinary creatinine to account for the influence of dilution on biomarker concentration (65). Additional details regarding assays, including coefficients of variation, are provided in Supplemental Table 7.

#### Data collection and validation.

Data for the primary and key secondary endpoints were collected on a daily basis for the first 7 days following randomization (Figure 2C). Study personnel collected detailed data by manual chart review and entered them into REDCap, a secure, web-based application (66), using a standardized case report form. Additional details regarding data collection, along with data quality and validation methods, are outlined in the Supplemental Methods.

### Transcriptomic analysis of monocytes

#### PBMC isolation.

PBMCs were isolated from blood samples (18mL) collected in EDTA-containing vacutainers on days 0 and 2 (Figure 2B). Isolation of PBMCs was performed using SepMate tubes (Stemcell Technologies) as per the manufacturer’s protocol. Following isolation, PBMCs underwent a controlled rate of freezing in 10% DMSO-containing cryopreservation medium, with long-term storage in liquid nitrogen.

#### Cell sorting by flow cytometry.

CD14^+^ monocytes were sorted from PBMCs by flow cytometry from patients (*N* = 131) and healthy donor controls (*N* = 11) using similar methods as we described previously (67). The cell sorting scheme is described in detail in the Supplemental Methods. We focused on monocytes, as their gene expression profile undergoes dynamic changes in critical illnesses like sepsis (68), respiratory failure (69), and cardiac surgery (70). Further, monocytes express the cellular machinery, such as the vitamin D receptor and CYP27B1, required to respond to calcifediol and calcitriol (39–42). These vitamin D metabolites have reliable immunomodulatory effects on monocytes in vitro (25, 71), but their effect on the immunophenotype of monocytes in critically ill patients has not been rigorously examined.

#### Bulk RNA-Seq analysis.

Monocytes were processed for RNA extraction, library preparation, and sequencing as a single batch per the standard operating protocol for the SmartSeq2 low input bulk RNA-Seq pipeline at the Broad Institute, as we described previously (72). Bulk RNA-Seq data were analyzed using the WARDEN workflow (67, 73). FastQ files were mapped to the reference genome (GRCh37) via the RNA-Seq aligner, STAR. The average mapping rate was 89.32%, with 10.14 million mapped reads per sample (additional details provided in the Supplemental Methods).

#### Prespecified genes.

We first analyzed 16 prespecified genes of interest that play a critical role in vitamin D metabolism (e.g., *VDR*, *CYP27B1*, *CYP24A1*) or downstream activity (e.g., *CAMP*), including those with antiinflammatory and cytoprotective effects relevant to acute organ injury prevention and treatment (e.g., *IL-10*, *HMOX1*) (Supplemental Table 5). For each of these targets, we examined the fold-change in monocyte gene expression from day 0 to 2, comparing the effect of calcifediol versus placebo and calcitriol versus placebo.

#### Global transcriptomics and GSEA.

Next, we identified genes with significantly different expression between healthy donors versus patients (using day 0 samples) (adjusted *P* < 0.05, absolute log_2_ fold change > 1). A total of 906 genes were differentially expressed. For each of these genes, we then examined the fold-change in their expression from day 0 to 2, comparing the effect of calcifediol versus placebo and calcitriol versus placebo. Finally, we performed gene set enrichment and pathway analyses on ranking estimated effects for all included features using the Molecular Signature Database for gene annotations, including Hallmark gene sets (additional details provided in the Supplemental Methods) (74).

### Statistics

Gene set enrichment and pathway analyses were performed on ranking estimated effects for all included features using the R package ClusterProfiler (75). A quasilikelihood F-test, implemented in the edgeR package (76), was used for differential expression of genes. Multiple comparisons were adjusted by the *q* value package in R, which uses an extension of the Benjamini-Hochberg method (Bioconductor, https://github.com/StoreyLab/qvalue; commit ID e861423).

All analyses were conducted on an intention-to-treat basis. We determined that a sample size of 50 patients per group (*N* = 150 total) would provide 80% power to detect a 0.62 standard deviation difference in time-averaged SCr levels in each of the active treatment groups versus placebo, with a 2-sided alpha of 2.5% to account for 2 active treatment groups. To evaluate the primary endpoint, we compared the ranks between each of the active treatment groups versus the placebo group using the Wilcoxon rank-sum test.

To evaluate secondary endpoints, we used Fisher’s exact and χ^2^ tests for binary variables and the Wilcoxon rank-sum test for continuous variables. Differences between groups in plasma 25D and 1,25D levels on day 0 were assessed with the Wilcoxon rank-sum test. To assess differences between groups in longitudinal parameters (e.g., 25D, 1,25D, calcium, phosphate, and SOFA score), we used mixed linear models for repeated measures.

Missing data were not imputed for primary or secondary clinical endpoints, and the amount of data missing for each variable is provided in Tables 1 and 2 and Supplemental Table 3. For longitudinal parameters, we used multiple imputation by chained equations to impute missing data, with 5 complete datasets created and results pooled using Rubin’s rules.

All comparisons are 2 tailed, with *P* < 0.025 considered significant for the primary endpoint to account for 2 active treatment groups, and *P* < 0.05 considered significant for secondary endpoints. Secondary endpoints were considered hypothesis-generating; therefore, no adjustment for multiplicity was performed. Analyses were performed using SAS Version 9.4 (SAS Institute Inc.).

### Study approval

Patients or their surrogates provided written informed consent. All protocols were approved by the Mass General Brigham Institutional Review Board (protocol nos. 2016P002527 and 2019P003592). The trial was registered at ClinicalTrials.gov (NCT02962102).

### Data availability

Values for all data points shown in graphs in the main manuscript and supplemental material are provided in the online Supporting Data Values file. Deidentified human subject data are available upon reasonable request from the corresponding author. RNA-Seq data are deposited at NCBI’s Gene Expression Omnibus (GEO) (GSE300480). This paper does not report original code. Any additional information required to reanalyze the data reported in this paper is available from the corresponding author upon reasonable request.

## Author contributions

DEL and SSW designed the study. DEL acquired funding, obtained an IND application to conduct the study, consented all patients, and wrote the initial draft of the manuscript. TS and SG assisted with preparation of figures, tables, and data analysis. KZ assisted with investigational product development and study design. JAD conducted the statistical analyses. The monocyte transcriptomics study was designed and executed by TT, CC, HA, and EYK and was supervised by EYK. EYK drafted the related sections of the manuscript, which were edited by TT, CC, and DEL. DSA and AAG assisted with preparation of figures and tables and data analysis. All authors critically revised and approve of the final manuscript.

## Funding support

This work is the result of NIH funding, in whole or in part, and is subject to the NIH Public Access Policy. Through acceptance of this federal funding, the NIH has been given a right to make the work publicly available in PubMed Central.

NIH/NIDDK grant K23DK106448 (to DEL).

NIH/NHLBI R01HL16687 (to EYK).

## Supplementary Material

Supplemental data

ICMJE disclosure forms

Supporting data values

## Figures and Tables

**Table 3:**
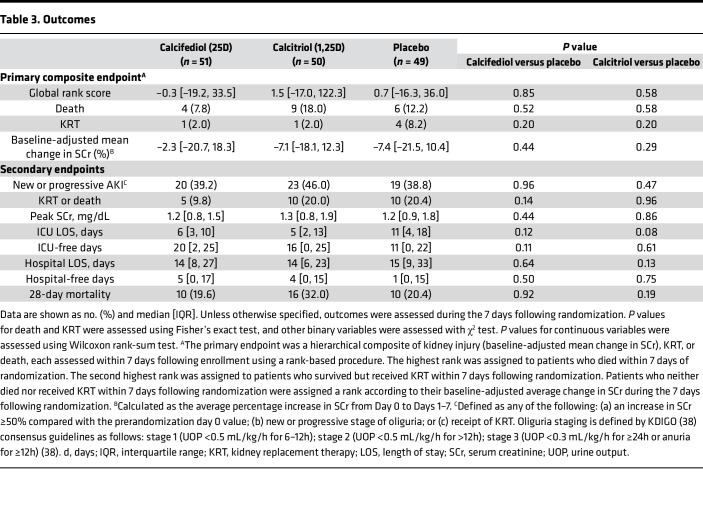
Outcomes

**Table 2:**
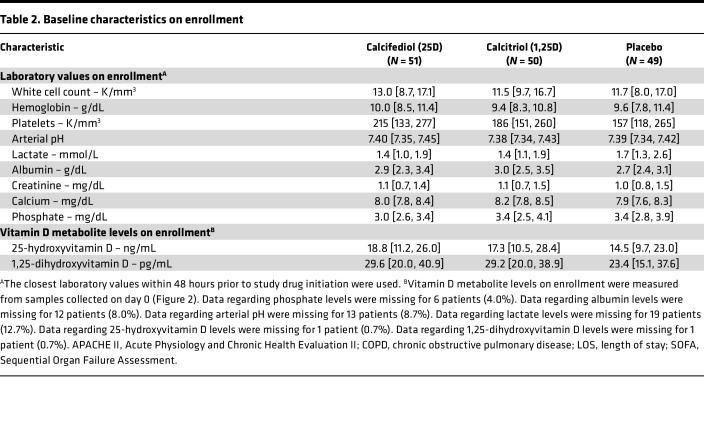
Baseline characteristics on enrollment

**Table 1:**
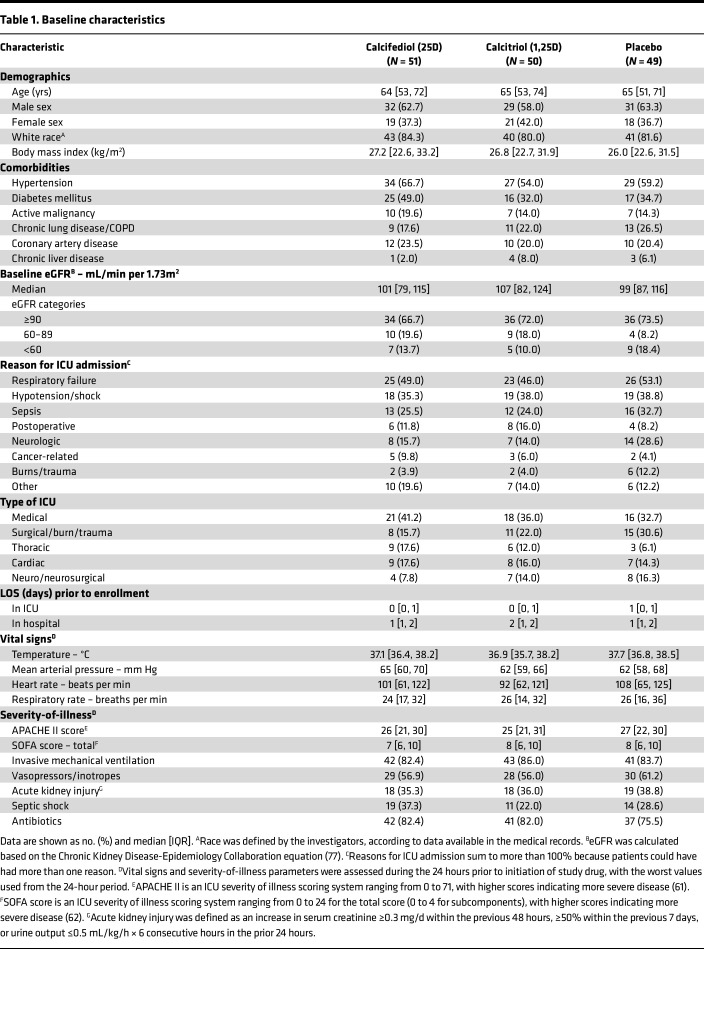
Baseline characteristics

## Version 1 09/09/2025

In-Press Preview

## Version 2 10/22/2025

Electronic publication
