# Supplementary material for: Randomized trial of activated vitamin D for acute kidney injury prevention in critically ill patients
Source: JCI Insight. 2025 Sep 9;10(20):e193523. doi: 10.1172/jci.insight.193523 (PMC12581674; doi:10.1172/jci.insight.193523)
Supplement: Supplemental data [file jciinsight-10-193523-s216.pdf]

BRIGHAM HEALTH
BRIGHAM AND WOMEN'S HOSPITAL

HARVARD MEDICAL SCHOOL

ACTIVATE-AKI: Activated Vitamin D for the Prevention and Treatment of Acute Kidney Injury

Detailed Protocol
December 25, 2018

Primary Investigators:

David E. Leaf, MD, MMSc
Assistant Professor of Medicine, Harvard Medical School
Associate Physician, Division of Renal Medicine, Brigham and Women's Hospital

Sushrut S. Waikar, MD, MPH
Associate Professor of Medicine, Harvard Medical School
Constantine L. Hampers, MD Distinguished Chair in Renal Medicine
Director of Translational Research and Ambulatory Services, Division of Renal Medicine, Brigham and Women's Hospital

TABLE OF CONTENTS

ABBREVIATIONS……3
TRIAL SUMMARY……4
I. BACKGROUND AND SIGNIFICANCE……5
A. Historical background……5
B. Previous pre-clinical and clinical studies supporting the research……6
C. Justification of agents……7
D. Justification of dosing regimens……8
E. Safety data on 25D (calcifediol)……11
F. Safety data on 1,25D (calcitriol)……13
G. Rationale and potential benefits to patients/society……14
II. SPECIFIC AIMS……15
III. SUBJECT SELECTION……16
A. Inclusion and exclusion criteria……16
B. Source of subjects……16
IV. SUBJECT ENROLLMENT……17
A. Methods of enrollment……17
B. Procedures for obtaining informed consent……17
C. Treatment assignment and randomization……18
V. STUDY PROCEDURES……19
A. Parameters to be measured……19
B. Drugs to be used……20
C. Devices to be used……21
D. Procedures/surgical interventions……21
E. Data to be collected……21
F. Dosing modifications……21
G. Adaptive trial design……21
VI. BIOSTATISTICAL ANALYSIS……22
A. Specific data variables being collected for the study……22
B. Study end points……22
C. Statistical methods……22
D. Power analysis……22
VII. RISKS AND DISCOMFORTS……21
A. Complications of surgical and non-surgical procedures……23
B. Drug side effects and toxicities……23
C. Confidentiality……25
D. Device complications/malfunctions……25
E. Psychosocial risks……25
F. Radiation risks……25
VIII. POTENTIAL BENEFITS……26
A. Potential benefits to participating individuals……26
B. Potential benefits to society……26
IX. MONITORING AND QUALITY ASSURANCE……27
A. Independent monitoring of source data……27
B. Safety monitoring……27
C. Outcomes monitoring……27
D. Adverse event reporting……27
X. REFERENCES……28

ABBREVIATIONS

1,25D = 1,25-dihydroxyvitamin D
25D = 25-hydroxyvitamin D
AKI = Acute Kidney Injury
APACHE = Acute Physiology and Chronic Health Evaluation
AUC = Area Under the Curve
BWH = Brigham and Women's Hospital
CKD = Chronic Kidney Disease
DSMB = Data safety and monitoring board
HED = Human Equivalent Dose
ICU = Intensive Care Unit
ESRD = End Stage Renal Disease
MCT Oil = Medium Chain Triglyceride Oil
NIDDK = National Institute of Diabetes and Digestive Kidney Diseases
NIH = National Institutes of Health
PBMC = Peripheral Blood Mononuclear Cells
PHRC = Partners Human Research Committee
RCT = Randomized Controlled Trial
RRT = Renal Replacement Therapy
SCr = Serum creatinine
SOFA = Sequential Organ Failure Assessment
ULN = Upper limit of normal

TRIAL SUMMARY

Title
Activated Vitamin D for the Prevention and Treatment of Acute Kidney Injury (ACTIVATE-AKI)

Objective
To assess the efficacy of calcifediol (25-hydroxyvitamin D [25D]) and calcitriol (1,25-dihydroxyvitamin D [1,25D]) in preventing and reducing the severity of acute kidney injury (AKI) in critically ill patients.

Hypothesis
Compared to placebo, treatment with calcifediol or calcitriol will reduce the occurrence and severity of renal injury, as determined by a composite of time-averaged daily serum creatinine (SCr) levels for 7 days, renal replacement therapy (RRT), and death.

Study Design
ACTIVATE-AKI is a 3-arm, randomized, double-blind, placebo-controlled, phase II trial (n=150) of vitamin D metabolites in critically ill patients at risk of severe AKI. We will randomly assign participants in a 1:1:1 fashion to receive calcifediol, calcitriol, or placebo. Study drugs will be administered on a daily basis for 5 days, and will be administered either orally or via naso/orogastric tube.

Study Sites
ACTIVATE-AKI is a single-center study that will recruit patients from intensive care units at Brigham and Women's Hospital (BWH).

Funding
ACTIVATE-AKI is approved and funded by the National Institutes of Health (NIH) / National Institute of Diabetes and Digestive Kidney Diseases (NIDDK) grant K23DK106448 (to David Leaf, MD).

Registration on clinicaltrials.gov
NCT02962102

I. BACKGROUND AND SIGNIFICANCE

A. Historical background

Overview of AKI
Acute kidney injury (AKI) is associated with markedly increased hospital length of stay, mortality, and cost.[1-3] Despite years of investigation, development of therapies for the prevention and treatment of AKI have been largely unsuccessful. Therefore, investigation of novel therapeutic targets is needed.

Overview of vitamin D metabolism
Vitamin D is derived from dietary sources and sunlight-induced cutaneous synthesis, and is converted to 25-hydroxyvitamin D (25D) in the liver (**Figure 1**).[4] 25D is converted to its biologically active form, 1,25-dihydroxyvitamin D (1,25D), in the kidneys and other tissues by 1-α hydroxylase.[5] The conversion of 25D to 1,25D is tightly regulated by two opposing hormones: parathyroid hormone (PTH), which stimulates 1-α hydroxylase, and fibroblast growth factor-23 (FGF23), which inhibits 1-α hydroxylase. FGF23 also stimulates 24-hydroxylase, which inactivates both 25D and 1,25D.[6]

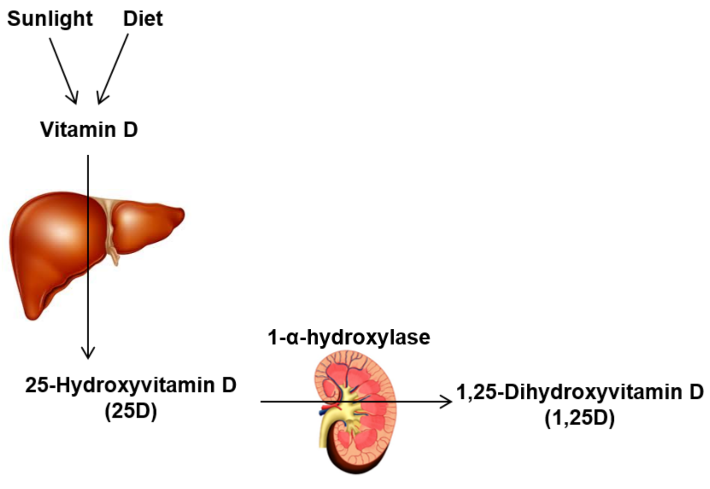

Figure 1. Vitamin D Metabolism

Non-classical actions of 1,25D
In addition to maintaining calcium and phosphate homeostasis, a variety of non-classical effects of vitamin D metabolites are also appreciated. 1,25D binds to the intracellular vitamin D receptor (VDR), which is expressed nearly ubiquitously.[7] This complex then translocates into the nucleus where it binds to specific DNA sequence elements in vitamin D-responsive genes, ultimately influencing the expression of over 200 target genes.[8] These target genes affect a variety of critical physiologic functions relevant to AKI, including immune/inflammatory pathways and vascular endothelial cell activation.[9]

Interventional studies of vitamin D metabolites for AKI prevention in humans
Despite promising data on vitamin D metabolites as therapeutic agents in animal models of AKI (summarized below), as well as epidemiologic studies from our group[10] and others[11-13] suggesting that deficiencies in vitamin D metabolite levels contribute to mortality in critical illness, no interventional study has evaluated vitamin D metabolites as novel therapeutic agents for AKI prevention/treatment in humans. We recently published the results of a pilot randomized controlled trial (RCT) evaluating the immunomodulatory effects of a single 2 µg IV dose of calcitriol (1,25D) in critically ill patients with severe sepsis.[14] We found that calcitriol led to increased leukocyte mRNA expression of IL-10 and HO-1, which have important antiinflammatory effects and prevent AKI in animal models.[15-19] The current study will expand on these findings by comprehensively evaluating the renoprotective effects of two vitamin D metabolites – calcifediol (25D) and calcitriol (1,25D) – in critically ill patients at risk of AKI.

B. Previous pre-clinical and clinical studies supporting the proposed research

Overview

The "non-classical" effects of vitamin D metabolites on inflammation have been demonstrated in numerous *in vitro* studies. Further, the beneficial effects of vitamin D metabolites on AKI prevention have been demonstrated in numerous animal models. Finally, extensive safety data on vitamin D metabolites are available from clinical trials in humans.

In vitro studies of 1,25D and inflammation

In vitro studies have demonstrated potent antiinflammatory effects of 1,25D. Treatment of T lymphocytes, monocytes, and other cells with 1,25D downregulates proinflammatory cytokines (e.g., IL-2, IL-6, TNF-α),[20-25] upregulates antiinflammatory cytokines/enzymes (e.g., IL-10, heme oxygenase-1 [HO-1]), [26-31] and increases the frequency of antiinflammatory T regulatory cells (Tregs).[32-34] Upregulation of IL-10, HO-1, and Tregs protect against AKI in animal models.[15-19, 35-37]

1,25D and AKI in animal models

In rodents, 1,25D attenuates renal injury induced by a wide variety of insults/toxins including ischemia,[38-40] gentamicin,[41] cyclosporine,[42] cisplatin,[43] glomerulonephritis,[44] and obstruction.[45]

Clinical studies of vitamin D metabolites in non-critically ill patients

Numerous RCTs have evaluated vitamin D metabolites as therapeutic agents across a wide variety of clinical settings, and are reviewed elsewhere as meta-analyses.[46-49] While these studies have reported mixed findings with respect to efficacy, they have demonstrated a very favorable safety profile (discussed further below under sections on safety).

Clinical studies of vitamin D metabolites in critically ill patients

A recently published RCT evaluated the effects of a large (540,000 I.U.) oral dose of vitamin D versus placebo among 492 critically ill patients. While the study found no effect on the primary outcome of hospital length of stay, a pre-specified subgroup analysis conducted in patients with severe vitamin D deficiency (defined as plasma 25D levels < 12 ng/ml) found a significant reduction in hospital mortality.[50] Importantly, no change in mean total serum calcium levels was observed in the vitamin D versus placebo groups at the two follow-up time points (days 3 and 7) where it was tested, and only a single patient in the vitamin D group developed hypercalcemia, with a total serum calcium level of 12.0 mg/dl.

Preliminary data from our pilot RCT of calcitriol

We recently published the results of a pilot RCT of calcitriol (1,25D).[14] We randomly assigned 67 critically ill patients with severe sepsis to receive either a single 2 µg IV dose of calcitriol or placebo. Although the primary endpoint of plasma cathelicidin levels was null, patients randomized to calcitriol had higher IL-10 (P=0.03) and HO-1 (P=0.004) leukocyte mRNA expression at 24h. Additionally, we found a graded relation between change in 1,25D levels and change in IL-10 (P=0.004) and HO-1 (p<0.001) expression. No hypercalcemia or other adverse events were observed.

C. Justification of agents

25D vs. 1,25D

Despite promising data on the antiinflammatory and renoprotective effects of 1,25D in preclinical models, it is unknown whether administration of vitamin D metabolites to critically ill patients will have beneficial immunomodulatory effects, and whether these effects will translate into improved clinical outcomes. Additionally, it is unknown whether 1,25D or its precursor, 25D, will have greater efficacy in humans. While 1,25D is the metabolite used in most preclinical studies, it is unclear whether the concentrations of 1,25D used *in vitro* (1 to 100 nM in most studies) can be achieved in humans. Additionally, although 1,25D is the "active" metabolite of vitamin D, it circulates in the plasma at ~1,000-fold lower concentrations than 25D. Target cells such as monocytes express the 1-α-hydroxylase enzyme that converts 25D into 1,25D,[51, 52] and are therefore capable of converting local 25D into 1,25D. Indeed, the ability of circulating 1,25D to exert immunomodulatory effects in humans has been questioned,[53] and some experts have advocated targeting 25D rather than 1,25D to facilitate local endogenous 1,25D production in target cells.[53-56] The current 3-arm study will address these uncertainties by evaluating the effects of both calcifediol (25D) and calcitriol (1,25D) versus placebo.

Overview of 25D

Plasma 25D levels can be raised indirectly by administering its precursor, vitamin D, or directly by administering 25D itself (**Figure 1**). A disadvantage of administering the precursor, vitamin D, is that it requires several days to saturate adipose tissue stores and raise plasma 25D levels to the low-normal range (30-35 ng/ml).[55, 57] A recently published RCT evaluated the effects of a large (540,000 I.U.) enteral dose of vitamin D versus placebo among 492 critically ill patients, and found no effect on hospital length of stay, the primary outcome.[50] In this study, only ~50% of the patients in the vitamin D arm actually achieved target plasma 25D levels >30 ng/ml by day 3. In contrast, administration of oral 25D raises plasma 25D levels within hours.[58] Thus, in the current study we will administer 25D.

FDA status of 25D (calcifediol)

Calderol. 25D was formerly available for use in patients as "Calderol". Calderol was approved by the FDA in 1980 for the treatment of hypocalcemia in patients undergoing chronic renal dialysis. Calderol was withdrawn from the market in 2002 for commercial reasons which were not associated with safety or efficacy of the product.

Rayaldee. 25D is currently FDA-approved as "Rayaldee" for the treatment of vitamin D deficiency in chronic kidney disease stage 3 or 4 patients with secondary hyperparathyroidism.

Overview of 1,25D

The rationale for evaluating 1,25D as a therapeutic agent in AKI is three-fold: 1) the renoprotective effects of vitamin D metabolites in animal models of AKI were demonstrated exclusively with 1,25D; 2) there may be impaired renal[59] and extra-renal[60] conversion of 25D to 1,25D in critical illness due to elevated FGF23[61] or other mechanisms; 3) our preliminary data from a pilot RCT in critically ill patients with sepsis demonstrate that a single 2 µg IV dose of 1,25D increases leukocyte mRNA expression of IL-10 and HO-1,[14] which are important antiinflammatory proteins that prevent AKI in animal models.

FDA status of 1,25D (calcitriol)

1,25D is available for use in patients as calcitriol, and is currently FDA-approved for the treatment of hypocalcemia in patients undergoing chronic renal dialysis.

D. Justification of dosing regimens

Overview of dosing regimens

Dosing regimens are shown in **Figure 2**. Participants assigned to 25D will be administered a liquid formulation of calcifediol, either orally or via naso/orogastric tube, at a dose of 400µg on day 0 (the day of enrollment) and 200µg on days 1, 2, 3, and 4. Participants assigned to 1,25D will be administered a liquid formulation of calcitriol, either orally or via naso/orogastric tube, at a dose of 4µg daily on days 0, 1, 2, 3, and 4. Participants assigned to placebo will be administered medium chain triglyceride (MCT) oil.

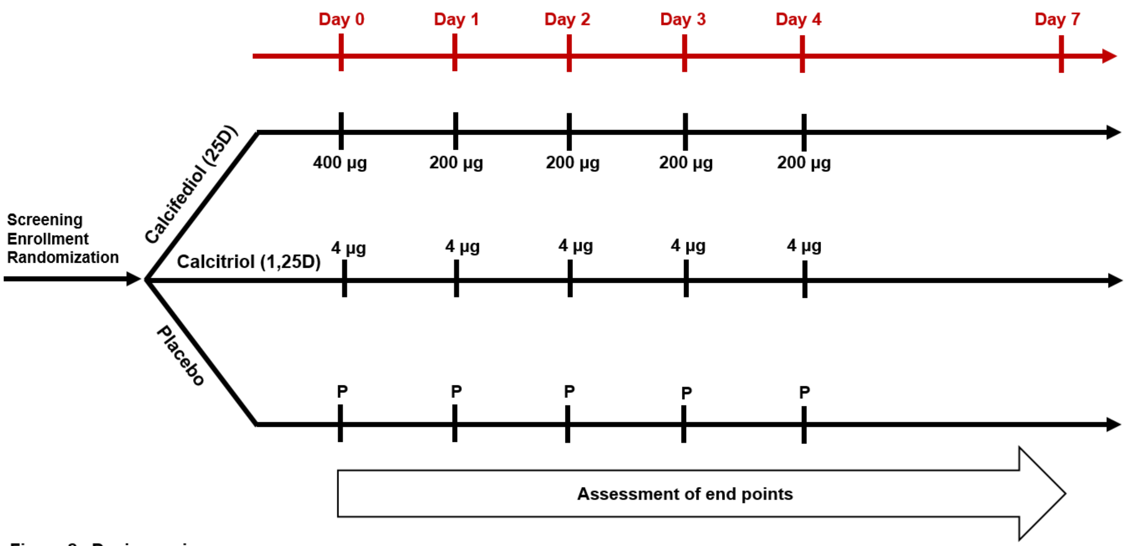

Figure 2. Dosing regimens

Rationale for dose of 25D

The dosing regimen of calcifediol in the current study is intended to achieve plasma 25D levels > 50 ng/ml throughout the majority of the first 7 days of the study. While optimal plasma 25D levels in critically ill patients are unknown, epidemiologic studies in this patient population have consistently shown that lower levels are associated with adverse clinical outcomes.[11-13, 62] A study conducted in healthy volunteers found that raising plasma 25D levels to 55 ng/ml with administration of oral vitamin D resulted in a significant increase in the frequency of regulatory T cells (Tregs),[63] which have an important protective role in AKI.[64] Additionally, a recently published RCT conducted in critically ill ventilated patients found that raising 25D levels to 55 ng/ml resulted in decreased hospital length of stay.[65]

Rationale for dose of 1,25D (data from in vitro studies)

Most *in vitro* studies demonstrating immunomodulatory effects of calcitriol used concentrations ranging between 1 to 100 nmol/L.[8, 31, 33, 66-68] In our recently completed pilot RCT evaluating the immunomodulatory effects of a single 2 µg intravenous dose of calcitriol among 67 critically ill patients, maximum mean (SD) plasma 1,25D levels were 0.2 (0.1) nmol/L,[14] substantially lower than concentrations used in vitro, which may account for the largely negative findings in the study. Thus, higher doses of calcitriol – which are nonetheless still substantially lower than doses that have been used safely in prior humans studies (discussed further in section F below) – are planned in the current study. Additionally, repeated dosing is planned (**Figure 2**) to account for the short half-life of calcitriol.

Rationale for dose of 1,25D (data from animal studies)

Animal models of AKI prevention with calcitriol

- Xu et al[69] evaluated the effects of calcitriol pretreatment on renal oxidative stress in an LPS model of sepsis-induced AKI in mice. Mice were pretreated with three oral doses of calcitriol at a dose of 25 µg/kg at 1, 24, and 48 hours before LPS injection. Calcitriol treatment compared to placebo resulted in significant attenuation of LPS-induced renal oxidative stress determined by markers of lipid peroxidation, renal inducible nitric oxide synthase expression, and other measures. The human equivalent dose (HED) of calcitriol is shown below in **Table 1**.

- Kapil et al[70] subjected rats to 40 minutes of bilateral renal ischemia-reperfusion injury (IRI). Prior to IRI, calcitriol was administered intraperitoneally on a daily basis for 7 days at the following doses: 0.25 µg/kg, 0.5 µg/kg, and 1 µg/kg. Treatment with each of these calcitriol dosing regimens compared to control-treated animals significantly attenuated the effect of IRI on kidney function, with the maximum beneficial effect observed in the group that received calcitriol 0.5 µg/kg. The HED of calcitriol in shown below in **Table 1**.

- Sezgin et al[40] subjected rats to a combination of right-sided nephrectomy plus left sided IRI. Rats were pretreated with calcitriol 0.5 µg/kg intraperitoneally on a daily basis for 7 days proir to renal IRI. Treatment with calcitriol compared to control-treated animals had a significant beneficial effect on biochemical parameters such as serum creatinine and blood urea nitrogen, as well as renal tissue expression of glutathione, superoxide dismutase, and nitric oxide activity. The HED of calcitriol in shown below in **Table 1**.

Animal models of autoimmune disease prevention/treatment with calcitriol

- Mathieu et al[71] evaluated the ability of calcitriol to prevent autoimmune diabetes in non-obese diabetic (NOD) mice. NOD mice received calcitriol 5 µg/kg intraperitoneally every other day beginning at the age of 21 days and ending at the age of 200 days or on the day diabetes was diagnosed. Calcitriol compared to placebo resulted in significant prevention of diabetes. The HED of calcitriol in shown below in **Table 1**.

- Branisteanu et al[72] evaluated the efficacy of calcitriol in preventing autoimmune encephalomyelitis in mice. Calcitriol at a dose of 5 µg/kg intraperitoneally every 2 days was effective at preventing paralysis in 70% of treated mice. The HED of calcitriol in shown below in **Table 1**.

Reference	Animal Model	Calcitriol Dose	HED	HED in a 100 kg individual
Xu et al[69]	Sepsis-induced AKI in mice	25 µg/kg	2.0 µg/kg	203 µg
Kapil et al[70]	Renal IRI in rats	0.5 µg/kg	0.1 µg/kg	8.1 µg
Sezgin et al[40]	Unilateral nephrectomy + renal IRI in rats	0.5 µg/kg	0.1 µg/kg	8.1 µg
Mathieu et al[71]	Autoimmune diabetes in NOD mice	5 µg/kg	0.4 µg/kg	40.7 µg
Branisteanu et al[72]	Autoimmune encephalomyelitis in mice	5 µg/kg	0.4 µg/kg	40.7 µg
				**Average calcitriol dose = 60.1 µg**<br><br>**Median calcitriol dose = 40.7 µg**

Table 1. Summary of animal studies in which calcitriol exerted protective immunomodulatory effects. HED is calculated by applying a conversion factor (6.2-to-1 for rats-to-humans; 12.3-to-1 for mice-to-humans) to account for differences in metabolism between rodents versus humans. Abbreviations: AKI, acute kidney injury; HED, human equivalent dose; IRI, ischemia reperfusion injury; NOD, non-obese diabetic.

E. Safety data on 25D (calcifediol)

Overview
The only known toxicity of vitamin D metabolites, including calcifediol (25D) and calcitriol (1,25D), is hypercalcemia. The dosing regimens of calcifediol planned in the current study are very unlikely to produce hypercalcemia because they are lower than doses that have been used safely in prior studies in both animals and humans.

Animal studies
In mice, doses of 25D required to induce hypercalcemia are 1mg/kg orally on a daily basis for 5 weeks.[73] In contrast, the highest dose of calcifediol in the current proposal is 400 µg, or 0.004 mg/kg in a 100 kg adult. Thus, we propose a dosing regimen that is approximately 200-fold lower than doses which induce hypercalcemia in mice. Further, we propose a much shorter duration of administration (5 doses over 5 days, in contrast to daily dosing for 5 weeks).

Human studies
In a study conducted in 27 healthy adult volunteers, oral calcifediol was administered at doses of 1.5 µg/kg, 5 µg/kg, and 10 µg/kg of body weight. Thus, a 70 kg individual assigned to the highest group would have received a 700 µg dose of calcifediol. No hypercalcemia nor any other adverse effects were observed.[58] In the same study, a single 1,000 µg IV dose of calcifediol was administered to two healthy volunteers and did not result in hypercalcemia or any other adverse events.[58]

In a separate study conducted in 12 healthy adult volunteers, oral calcifediol was administered at 10 µg/kg of body weight. No hypercalcemia nor any other adverse effects were observed. Mean plasma calcium levels rose only minimally from 9.5 mg/dl to 9.7 mg/dl at 24 hours.[74]

In a study conduced in elderly nursing home patients, 22 patients were assigned to received a single dose of calcifediol 10 µg/kg of body weight. No hypercalcemia nor any other adverse effects were observed. No significant differences were observed in serum calcium levels in calcifediol-treated versus control patients.[75]

In a more recent study conducted in 29 subjects with stage 3 or 4 chronic kidney disease, subjects were randomly assigned to receive a single dose of oral calcifediol 450 µg, a single dose of oral calcifediol 900 µg, or an intravenous dose of calcifediol 448 µg. No significant increases in serum calcium were observed in any treatment group during the post-dose period.[76]

The safety of repeated (daily) dosing of oral calcifediol in humans has been demonstrated in multiple smaller studies[77-79] as well as in two phase III RCTs, which included a total of 429 subjects.[80] In the latter studies, patients received 30 or 60 µg of 25D on a daily basis for 26 weeks. Subjects assigned to 25D had an inconsequential increase in mean serum total calcium levels (0.2 mg/dl versus 0.1 mg/dl in the placebo group), and the percentage of patients who experienced at least one treatment-emergent adverse event was similar in the 25D versus placebo groups.[80] Only six subjects (2%) in the calcifediol group required dose reductions for protocol-defined hypercalcemia (2 consecutive serum calcium values > 10.3 mg/dl).

The current study will administer a total of five enteral doses of calcifediol over 5 days. The rationale for this approach is that 25D levels are known to decline by ~50% of peak levels by 24 hours.[58, 74] To minimize the potential for a stepwise increase in plasma 25D levels with repeated dosing, the first dose will be 400 µg, and subsequent doses will be 200 µg (**Figure 2**).

Summary

Plasma levels of 25D considered "toxic" (i.e. associated with hypercalcemia) have been reported to be in excess of 214 ng/ml.[81 82] Based on the known pharmacokinetics of calcifediol from the studies above, plasma 25D levels are very unlikely to ever approach this limit.

F. Safety data on 1,25D (calcitriol)

Overview
The only known toxicity of vitamin D metabolites, including calcifediol and calcitriol, is hypercalcemia. The dosing regimens of calcitriol planned in the current study are very unlikely to produce hypercalcemia because they are lower than doses that have been used safely in previous studies in humans.

Human studies of oral calcitriol
In a phase I dose-escalation pharmacokinetics study of oral calcitriol in patients with advanced malignancy, patients received calcitriol for 3 consecutive days each week. The highest dosing level of calcitriol administered was 38 µg. At this dose, the average increase in serum calcium was 0.5 mg/dl, and no dose-limiting toxicity occurred.[83]

In a phase II study of high dose oral calcitriol, 39 patients with prostate cancer were randomly assigned to receive calcitriol 0.5 µg/kg (e.g., 35 µg in a 70 kg adult) once per week for 4 weeks or placebo. No patients developed hypercalcemia, and no significant changes in serum calcium were detected. The median and range of serum total calcium levels at baseline was 9.3 (8.5 to 10.4) mg/dl, compared with 9.3 (8.6 to 10.1) mg/dl on day 3, 9.4 (8.7 to 10.2) mg/dl on day 10, and 9.3 (7.8 to 10.0) mg/dl on day 25. No significant differences in adverse events were reported between calcitriol vs. placebo-treated patients.[84]

In a phase I pharmacokinetics study of an oral calcitriol formulation, DN-101, 38 patients received a single-dose administration of 15, 30, 60, 75, 90, 105, 135, or 165 µg of DN-101. No hypercalcemia was observed at doses less than 60 µg, and the hypercalcemia that developed at higher doses (in 13.2% of patients) was grade 1 (minor and transient) in all cases.[85]

Human studies of intravenous calcitriol
In a phase I dose-escalation pharmacokinetics study of IV calcitriol in patients with advanced solid tumors, no patients developed hypercalcemia at weekly IV doses up to 15 µg. At higher weekly IV doses of 74 µg, only one of seven patients developed clinically relevant (grade 2 or higher) hypercalcemia, defined as serum calcium > 11.5 mg/dl.[86]

In a phase I pharmacokinetics study of IV calcitriol in combination with oral dexamethasone and gefitinib in patients with advanced solid tumors, 20 patients received IV calcitriol on weeks 1, 3, and weekly thereafter. The starting dose of calcitriol was 57 µg and escalation occurred in cohorts of three patients until the maximum tolerated dose was defined. Escalations included 74, 96, 125, and 163 µg doses. Doses of 125 µg were associated with grade 2 hypercalcemia (serum calcium 11.5-12.5 mg/dl) and doses of 163 µg were associated with two cases of grade 3 hypercalcemia (serum calcium 12.5-13.5). However, doses ranging from 57 to 96 µg did not produce any clinically relevant (grade 2 or higher) hypercalcemia (defined as serum calcium > 11.5 mg/dl).[87]

Summary
The dosing regimen of calcitriol planned in the current study (4 µg daily for 5 days) represents a dose far lower than dosing regimens that have been used safely in prior studies, and thus is very unlikely to cause hypercalcemia or other adverse events.

G. Rationale behind the proposed research, and potential benefits to patients/society

Despite compelling data from the pre-clinical and epidemiologic studies discussed above, it remains unknown whether administration of vitamin D metabolites (25D or 1,25D) to critically ill patients will have beneficial effects on acute kidney injury (AKI) or other clinically relevant outcomes. Finding an effect of vitamin D metabolites on AKI would represent a major advance in the field, especially since currently there are no drugs or interventions that reliably prevent or treat AKI in humans. Finding a beneficial effect of vitamin D metabolites on AKI in the current study would provide preliminary data to justify a larger interventional study of vitamin D metabolites among critically ill patients, which would be powered to detect hard clinical end points such as need for renal replacement therapy and death. Finally, the current study will answer important questions about vitamin D metabolism in critically ill patients, which will inform future interventional studies of vitamin D metabolites in this and other clinical settings.

II. SPECIFIC AIMS

We will enroll 150 critically ill patients at risk of severe AKI into a 3-arm, double-blind, randomized study of calcifediol (25D), calcitriol (1,25D), or placebo to test the following:

Primary Objective – Prevention of AKI incidence/severity
Test whether administration of calcifediol (25D) or calcitriol (1,25D), compared to placebo, will decrease the incidence/severity of AKI among ICU patients, assessed by daily measurement of SCr over 7 days.

Secondary Objectives – Urinary Injury and Immune/Inflammatory Biomarkers
Test the effects of calcifediol (25D) or calcitriol (1,25D), compared to placebo, on urinary injury biomarkers (NGAL and KIM-1) and key immune/inflammatory markers assessed by monocyte mRNA expression and flow cytometry.

Tertiary Objectives – Clinical Outcomes (exploratory)
Test the effects of calcifediol (25D) or calcitriol (1,25D), compared to placebo, on ICU- and hospital length of stay, mortality, duration of mechanical ventilation, and other clinical outcomes.

III. SUBJECT SELECTION

A. Inclusion and Exclusion Criteria

Inclusion Criteria	Exclusion Criteria
• Age ≥ 18<br>• Admitted to the ICU within 48h prior to enrollment<br>• Likely to remain in the ICU (alive) for ≥72h<br>• Naso/orogastric tube or ability to swallow<br>• High risk of severe AKI*	• Total serum calcium > 9.0 mg/dl or phosphate > 6.0 mg/dL within previous 48h<br>• Currently receiving oral calcium supplementation<br>• Ingestion of vitamin D_3 >1,000 IU/day or any 25D or 1,25D during the previous 7 days<br>• AKI stage 2 or 3 (based on SCr and/or UOP criteria)<br>• History of transplantation or chronically (>7days) receiving immunosuppressive medications (not including glucocorticoid steroids at a dose ≤ prednisone 20 mg/day)<br>• Neutropenia (<1000 PMNs/mm^3) in the previous 48h<br>• Primary parathyroid disease (active), granulomatous disease (active), or symptomatic nephrolithiasis in the previous 3 months<br>• Receiving cytochrome P450 inhibitors†<br>• CKD stage V or ESRD<br>• Hgb < 7 g/dL<br>• GI malabsorption<br>• Prisoner<br>• Pregnant or breastfeeding<br>• Enrolled in a conflicting study, including any study with a research blood draw

Table 2. Inclusion and Exclusion Criteria. *Patients are considered to be at high risk of severe AKI if they have either: AKI stage 1, defined as an increase in SCr ≥ 0.3 mg/d within the previous 48 hours, ≥ 50% within the previous 7 days, or urine output ≤ 0.5 ml/kg/h x 6 hours); or if they have an AKI risk score ≥6 – see Table 3. †Patients will be excluded if they are receiving any of the following medications: ketoconazole, atazanavir, clarithromycin, indinavir, itraconazole, nefazodone, nelfinavir, ritonavir, saquinavir, telithromycin, or voriconazole, since these drugs may inhibit enzymes involved in vitamin D metabolism.

Risk Factor	Points
Chronic	
Age>70y	1
Hypertension	1
Diabetes Mellitus	2
Chronic Liver Disease (cirrhosis, NAFLD, AIH, or chronic viral hepatitis)	2
Congestive Heart Failure (NYHA class 3 or 4)	2
Chronic Kidney Disease (baseline eGFR<60 ml/min/1.73m^2)	3
Acute	
Blood pH (venous or arterial) ≤ 7.30	2
Sepsis	2
Mechanical ventilation expected to last > 24 hours	2
Hypotension/shock, defined as MAP<70 mm Hg or use of pressors/inotropes	2

Table 3. AKI Risk Prediction Score

B. Source of Subjects

Subjects will be enrolled from the following intensive care units (ICUs) at Brigham and Women's Hospital (BWH): medical ICU (MICU), cardiac care unit (CCU), thoracic surgery ICU (TICU), surgical ICU (SICU), and neurology/neurosurgical ICU (NICU/NSICU).

IV. SUBJECT ENROLLMENT

A. Methods of enrollment

Patients from each of the ICUs listed above will be screened for eligibility, using electronic medical records, based on the inclusion/exclusion criteria listed above. Women of child-bearing potential will not be included without documentation of a negative pregnancy test, unless they have previously undergone hysterectomy or bilateral oophorectomy. Women of child bearing potential will be defined as any woman who has begun menstruation and not yet reached menopause. Menopause will be defined as any woman over the age of 45 who has not had a menstrual period for at least 12 months. For those patients who appear eligible based on electronic medical records, study investigators will confirm eligibility with the patient's nurse and attending physician of record.

B. Procedures for obtaining informed consent

Once permission for study enrollment has been obtained from the patient's attending physician, study investigators will request that a member of the patient's primary treating team (e.g., the intern, resident, fellow, attending, or nurse caring for the patient) ascertain the willingness of the patient to discuss the study with investigator David Leaf, M.D., investigator Sushrut Waikar, M.D, or investigator Finnian McCausland, M.D. Patients eligible and willing will then be approached by Dr. Leaf, Dr. Waikar, or Dr. McCausland to explain the rationale of the study, answer any questions, and ask for written informed consent to participate. Only physician-investigators will obtain informed consent. Subjects will be given up to 6 hours to consider participation given the time-sensitive nature of the study. The 6-hour period will begin once the patient or surrogate has received the consent form.

For patients deemed to lack decisional capacity by the primary treating team, study investigators will follow identical procedures as above with the patient's surrogate. The Partners Human Research Committee (PHRC) preferred order of surrogates will be followed, and the investigator will document the relationship of the surrogate to the subject in the research record. The order of surrogates will be:

> i) court appointed guardian with specific authority to consent to participation in research or authority to make health care decisions for a class of diagnostic and therapeutic decisions inclusive of the proposed research;
> ii) health care proxy/person with durable power of attorney with specific authority for making health care decisions inclusive of the proposed research; or
> iii) spouse, adult child, or other close family member who knows the subject well and has been involved in their care.

For patients deemed to lack decisional capacity who are enrolled by surrogate consent, assent of subjects will be required for participation in the research unless the subject is incapable of providing assent due to his/her medical condition (such as intubation/sedation). For patients deemed to lack decisional capacity who are enrolled by surrogate consent and who subsequently regain capacity during the study, investigators will explain the study (including updating them on what has happened so far as a result of being in the study), answer any questions, and ask for written informed consent to continue to participate. Alternatively, patients will have the option of withdrawing from the study, in which case all data and biological samples collected will be destroyed.

Although written consent from surrogates in person will be obtained whenever possible as the preferred means of consent for patients who are unable to consent, for patients who do not

have a surrogate available to provide written consent in person we will ask a member of the patient's primary treatment team (e.g., doctor or nurse) to contact the surrogate (using the same PHRC preferred order described above) by telephone to inform them of the study and to ascertain their willingness to speak with one of the M.D. study investigators. The primary team member making the phone call will verify the surrogate's name and relationship to the patient, and will be provided with a printed script containing bullet points for the call (script provided as separate attachment).

For surrogates expressing interest in hearing about the study, physician-investigator David Leaf, M.D., Sushrut Waikar, M.D., or Finnian McCausland, M.D., will first describe an overview of the study and answer any questions. If the surrogate is interested in hearing more about the study, the physician-investigator will ascertain whether the surrogate has plans of coming to the hospital in the next few hours, and will therefore be available to provide in person written consent. If the surrogate states that he/she is planning on coming to the hospital in the next few hours, the physician-investigator will complete the consent process in person. If the surrogate states that he/she is not planning on coming to the hospital in the next few hours, the physician-investigator will send the consent form to the surrogate electronically, either by secure email or facsimile. After confirming that the surrogate has a copy of the consent form in front of them, the physician-investigator will summarize the required elements of informed consent, review the consent form with the surrogate, answer any additional questions, and ask for written consent. If the surrogate agrees, the physician-investigator will ask the surrogate to sign and date the consent form and to send it back electronically by secure email or facsimile. Given the time-sensitive nature of the study, the surrogate will be asked to provide written consent as soon as possible and no longer than 6 hours from the time they receive the consent form.

Non-English speaking patients will be eligible for inclusion. We will use official Partners interpreters and the PHRC approved 'short form' consent document in the native language of the patient and/or their surrogate.

Using a checklist, we will document the following items of the informed consent process for each patient:

- If consent was from subject or surrogate
- If surrogate, in-person or on the phone
- If surrogate, relationship to subject
- If surrogate, could subject assent, and if not, why not?
- Was an interpreter required and short form used?
- If subject regains capacity to consent a later time; if so, document whether the subject provides written consent to continue to participate or not
- Copy of consent form provided to subject or surrogate/
- Copy of consent form uploaded into Epic?
- Research study associated to the patient in Epic?

C. Treatment assignment and randomization

Participants will be randomly assigned, in a 1:1:1 fashion, to receive calcifediol, calcitriol, or placebo. Randomization will be stratified by the presence or absence of AKI on enrollment, defined by an increase in serum Cr ≥ 0.3 mg/dl within the previous 48 hours, ≥ 50% within the previous 7 days, or urine output ≤ 0.5 ml/kg/h x 6 consecutive hours during any 6-hour block in the previous 24 hours. Randomization will be performed by the BWH research pharmacy using a computer generated sequence and permuted blocks.

V. STUDY PROCEDURES

A. Parameters to be measured

Clinical data
Upon enrollment in the study, the following demographic variables will be recorded: age, gender, race, ethnicity, comorbidities, active medications, active medical issues, reason for ICU admission, date of ICU/hospital admission, ICU location (e.g., MICU, CCU), and severity of illness at the time of study enrollment, as measured by APACHE II and SOFA scores.

In addition, the following clinical outcomes data will be recorded:

-ICU-, hospital-, and 28-day mortality
-ICU and hospital length of stay
-Incidence/severity of new/worsening AKI (defined by KDIGO guidelines)[88] within 1 week of enrollment
-Duration of mechanical ventilation
-Duration of vasopressor support
-Daily Sequential Organ Failure Assessment (SOFA) scores for the first 7 days of the study
-Physiologic data including heart rate, blood pressure, oxygenation, and urine output during the first 7 days of the study
-Hypocalcemia requiring I.V. calcium replacement during the first 7 days of the study
-Total serum calcium levels on a daily basis for the first 7 days of the study

28-day mortality
For patients discharged from the hospital prior to day 28, investigator David Leaf, M.D. will ascertain 28-day survival by calling the patient and/or their surrogate 28 days after enrollment as a courtesy follow-up to thank them for participating and to answer any questions they may have. The duration of the phone call is expected to last less than 2 minutes.

Blood samples
We will obtain blood samples on days 0, 1, 2, 3, 4, and 5. A summary of the volumes of blood that we will obtain at each time point is shown in **Figure 3**. On days 0 and 2 we will collect 18mL of blood, which will allow us to isolate peripheral blood mononuclear cells (PBMCs) and store them in liquid nitrogen for comprehensive analyses in the future, including flow cytometry and RT-PCR. 18mL of blood will ensure that we have a sufficient number of PBMCs to perform all of the planned analyses. Specifically, each mL of blood has approximately 10^6 PBMCs, and therefore 18mL of blood has approximately 18 x 10^6 PBMCs. We will aliquot and store the PBMCs as follows: aliquot #1 will have 10 x 10^6 PBMCs, and will be used for RT-PCR analyses on monocytes. Aliquots #2 and #3 will have 4 x 10^6 PBMCs each, and will be used for flow cytometry. For each of these analyses, the number of PBMCs being collected and archived represents the minimum amount necessary for the intended analyses.

In addition to the 18mL of blood on days 0 and 2 for PBMC isolation, we will also collect 2.5mL blood on days 0, 1, and 2 into PAXgene RNA tubes, which will allow us to evaluate leukocyte gene expression profiles. Finally, on days 1, 3, 4, and 5, we will collect 9mL of blood and store the plasma and DNA. 9mL of blood contains approximately 4.5mL of plasma (approximately 50% of whole blood is plasma), which will allow us to archive nine 500uL plasma aliquots at each time point. Collection of nine plasma aliquots at each time point will allow us to test the multiple hypotheses and measurements described below.

Thus, the total volume of blood that will be drawn over the course of the entire study, including PBMCs, DNA, RNA, and plasma, will be **79.5mL**. We will not enroll patients who are already enrolled in competing studies in which blood is being drawn, and we will not enroll patients with a hemoglobin level < 7 g/dl (**Table 2**). In most cases, we anticipate that blood samples will be obtained from central venous or arterial catheters by the patient's nurse. For patients who do not have a central venous or arterial catheter, blood will be obtained by venipuncture by a hospital phlebotomist, drawn concurrently with clinical blood samples whenever possible.

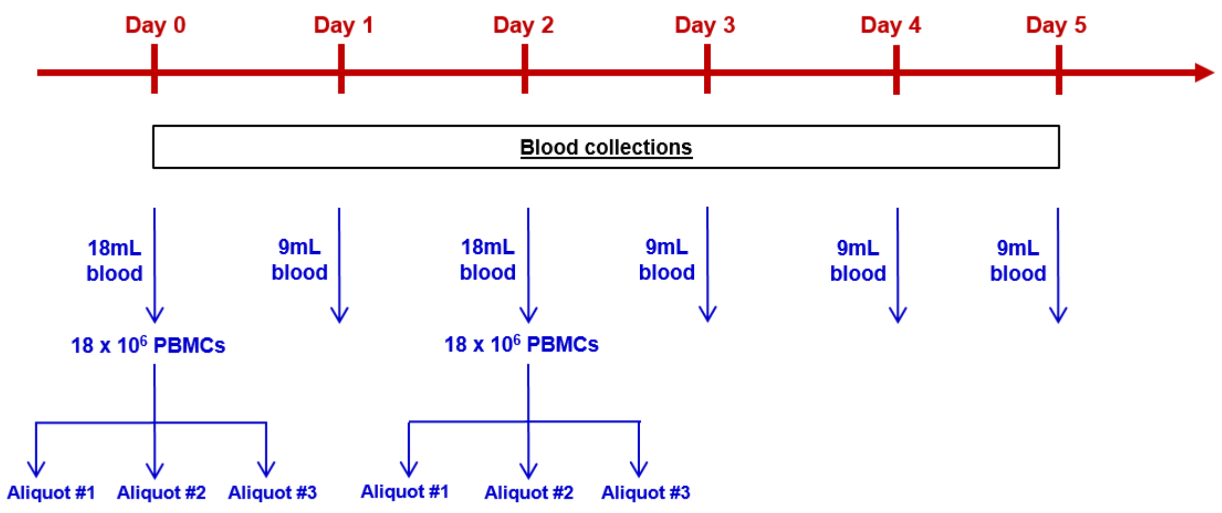

Figure 3. Blood Sample Collections. In addition to the volumes shown in the figure, 2.5mL blood will be collected into a PAXgene RNA tube on Days 0, 1, and 2.

The primary measurements that are anticipated to be performed on the plasma obtained from these collections will include: 25D, 1,25D, vitamin D-binding protein, heme oxygenase-1, interleukin-6, interleukin-10, thrombomodulin, parathyroid hormone, fibroblast growth factor-23, and cystatin C.

Urine
We will collect 30mL of urine at each of the above time points. In the majority of cases, urine will be collected from foley catheters placed for routine clinical care of ICU patients. Foley catheters will not be placed for research purposes. If a patient does not have a foley catheter in place for routine clinical care, urine will be collected using a bed pan, commode, or direct urination into a specimen cup. Anticipated tests that will be performed on the urine samples include neutrophil gelatinase-associated lipocalin (NGAL), interleukin 18, and kidney injury molecule-1 (KIM-1).

B. Drugs to be used

We will administer calcifediol, calcitriol, or placebo. The dosing regimens are provided in **Figure 2**. The rationale for these agents, justification of dosing regimens, and safety data are provided in sections 1C-F. Patients assigned to the placebo arm will receive medium chain triglyceride (MCT) oil, which has an identical appearance, color, consistency, and odor as calcifediol and calcitriol, both of which are also compounded in MCT oil.

Double-dummy design
Although calcifediol, calcitriol, and placebo will be identical to each other in appearance, the volumes administered will differ. For example, the concentration of calcitriol is 1 µg/ml, and therefore each 4µg dose will be administered in 4 ml. In contrast, the concentration of calcifediol is 100µg/ml. Since the first dose of calcifediol is 400µg, the first dose will also be administered in 4ml, identical to the calcifediol arm. However, calcifediol will be administered at a dose of 200µg on days 1, 2, 3, and 4 (**Figure 2**), which will be in 2ml. Therefore, to maintain blinding, we will use a double-dummy approach for drug administration on days 1, 2, 3, and 4 as follows:

Calcifediol Arm	Calcitriol Arm	Placebo Arm
Calcifediol 2ml<br>Placebo 4ml	Calcitriol 4ml<br>Placebo 2ml	Placebo 4ml<br>Placebo 2ml

C. Devices to be used
None

D. Procedures/surgical interventions
None

E. Data to be collected
The specific data variables that will be collected are provided in section V-A.

F. Dosing modifications
All patients enrolled will have total serum calcium levels monitored on a daily basis as part of routine clinical care in the ICU. All subsequent doses of study drug will be held for any patient who develops a total serum calcium level > 10.0 mg/dl after enrollment. Additionally, if at any time during the course of the study >10% of participants in either of the active treatment arms develops hypercalcemia, defined as per the BWH hospital laboratory as a total serum calcium level > 10.7 mg/dl, the dosing regimen for future participants assigned to that arm will be reduced by 25%. For the calcifediol arm, this 25% dose reduction would result in a new dosing regimen of 300 µg on day 0 and 150 µg on days 1, 2, 3, and 4. For the calcitriol arm, this 25% dose reduction would result in a new dosing regimen of 3 µg daily for 5 days. Unblinded safety data on hypercalcemia will be evaluated by the data and safety monitoring board (DSMB).

G. Adaptive Trial Design
After enrollment of the first 18 patients (6 patients in each of the three arms), we will stop enrollment to measure plasma 25D levels in the patients who received calcifediol to ensure that mean plasma 25D levels on days 1, 2, 3, 4, and 5 are > 50 ng/ml. If mean plasma 25D levels on days 1, 2, 3, 4, and 5 are < 50 ng/ml <u>and</u> the mean increase in serum total calcium levels from day 0 to day 5 is < 0.5 mg/dl, we will increase the dosing regimen for the calcifediol arm for the remaining study participants as follows:

Mean plasma 25D level on days 1 through 5	Calcifediol dose adjustment for remaining participants
40-50 ng/ml	500 µg on day 0 and 250 µg on days 1, 2, 3, and 4
<40 ng/ml	600 µg on day 0 and 300 µg on days 1, 2, 3, and 4

VI. BIOSTATISTICAL ANALYSIS

A. Specific data variables being collected for the study

The specific data variables that will be collected are provided in section V-A.

B. Study end points

Primary end point. The primary end point is a composite of renal injury, RRT, and death. Renal injury will be assessed by time-averaged (area under the curve [AUC]) levels of daily SCr for the first 7 days following enrollment. This end point has been used in previous clinical trials conducted in AKI,[89] and these measurements will be obtained through routine clinical testing. To account for missing data, we will standardize the AUC of daily SCr levels relative to the number of days in which SCr data are available. RRT and death are important confounders of SCr levels, as well as important end points by themselves, and will therefore be considered along with the AUC of SCr levels as part of the primary composite end point. RRT will be defined as receipt of any form of dialysis within the first 7 days of enrollment. Death will be defined as all-cause mortality within the first 7 days of enrollment.

To evaluate the primary composite end point, which includes both continuous (SCr levels) and dichotomous (RRT and death) variables, we will use a rank-based procedure, as prior studies have done,[90] as follows:

-Patients who die within 7 days of enrollment will be assigned the highest (worst) rank, with no difference in rank assigned to patients who die at different times within the first 7 days.
-Patients who survive >7 days but who require RRT within 7 days of enrollment will be assigned the second highest rank.
-All other patients will be assigned a rank based on the AUC of their SCr, as described above.

Secondary end points. Secondary end points will include new or worsening AKI (defined by KDIGO guidelines);[91] kidney function on hospital discharge (assessed by SCr); hospital- and ICU length of stay; and hospital-, ICU-, and 28-day mortality. We will also evaluate the AUC of urinary injury biomarkers, including KIM-1, NGAL, and IL-18, during the first 5 days using a similar global rank score strategy as described above. Finally, we will evaluate organ-failure free days (assessed by daily SOFA scores) during the first 7 days following enrollment. For the cardiovascular component of the SOFA score, we will used a modified scoring system that has improved accuracy in predicting outcomes,[92] and for the renal component we will assign a score of “4” (the highest score) to any participant who requires RRT.

C. Statistical methods

To evaluate the primary end point, we will compare the ranks between treatment groups versus the placebo group with the use of the Wilcoxon Rank Sum test. To evaluate secondary end points, we will compare means and medians between each of the active treatment groups versus the placebo group using parametric (e.g., t-test) and non-parametric (e.g., Wilcoxon Rank Sum) tests, as appropriate. To account for two active treatment groups, we will consider p values <0.025 to be significant for the primary end point. For secondary end points, all of which will be considered exploratory, we will consider p values <0.05 to be significant.

D. Power analysis

150 participants (N=50 per group) will provide >80% power to detect a 0.62 standard devation difference in the global rank score in each of the active treatment groups vs. placebo, with a two-sided α of 2.5%.

VII. RISKS AND DISCOMFORTS

A. Complications of surgical and non-surgical procedures

Not applicable

B. Drug side effects and toxicities

The only known toxicity of vitamin D metabolites, including calcifediol and calcitriol, is hypercalcemia.[81 82] Despite an excellent safety profile, transient hypercalcemia has been reported with calcitriol and, to a much lesser extent, with calcifediol. The risks of hypercalcemia in the current study are low, since the planned doses of calcifediol and calcitriol are lower than doses that have been used safely in prior studies in humans (discussed in detail in sections 1E and 1F). Additionally, <u>the following precautions will further minimize risk to patients:</u>

1) Limiting our study to patients with serum total calcium levels ≤ 9.0 mg/dl. Most prior clinical studies of calcifediol and calcitriol (discussed in sections 1E and 1F) were conducted in patients who were generally normocalcemic on enrollment. In contrast, most ICU patients have serum calcium levels in the low-normal range, and many patients (55.2% in one study)[93] are frankly hypocalcemic on arrival to the ICU. In the current study, we will exclude patients with serum total calcium levels > 9.0 mg/dl on enrollment (**Table 2**). By limiting enrollment to patients with serum total calcium levels ≤ 9.0 mg/dl, a substantial barrier exists for serum calcium levels to rise into the hypercalcemic range (>10.7 mg/dl) and an even larger barrier for clinically relevant hypercalcemia (>11.5 mg/dl).

2) Limited number of doses of study drug. In most prior clinical studies, calcifediol and calcitriol were administered on a regular (daily) basis for weeks to months. The current study will administer a total of only 5 doses of calcifediol or calcitriol drug over 5 days.

3) Close monitoring of total serum calcium levels. All patients enrolled will have total serum calcium levels monitored on a daily basis. Subsequent doses of study drug will be held in any patient with a serum calcium > 10.0 mg/dl.

4) Exclusion of patients with primary parathyroid disease or granulomatous disease. These conditions may be associated with upregulation of 1-α hydroxylase, which could increase the conversion of 25D to 1,25D and increase the risk of developing hypercalcemia. Additionally, we will exclude patients with symptomatic nephrolithiasis in the preceding 3 months, and those receiving oral calcium supplementation (**Table 2**).

5) Exclusion of patients receiving cytochrome P450 inhibitors. We will exclude patients receiving any of the following medications, since these drugs may inhibit enzymes involved in vitamin D metabolism and therefore could increase the risk of hypercalcemia: ketoconazole, atazanavir, clarithromycin, indinavir, itraconazole, nefazodone, nelfinavir, ritonavir, saquinavir, telithromycin, or voriconazole (**Table 2**).

6) Highly monitored setting. Patients will be in the highly-monitored setting of an ICU at a tertiary care academic hospital. Accordingly, if hypercalcemia or other adverse events occur they are likely to be appreciated immediately and appropriate resources will be readily available.

7) Pre-specified dose modification. As described in section V-F, if at any time during the course of the study >10% of participants in either of the active treatment arms develops hypercalcemia, defined by the BWH hospital laboratory as a total serum calcium level > 10.7 mg/dl, the dosing regimen for future participants assigned to that arm will be reduced by 25%.

8) DSMB. A DSMB will meet every 6 months to review unblinded data on all adverse events, with an emphasis on serum total calcium levels, and will submit a report to the IRB after each meeting. Additionally, data safety monitoring will be reviewed by investigators David E. Leaf, MD, and Sushrut S. Waikar, MD, on a weekly basis.

C. Confidentiality

Loss of confidential patient information is a potential risk in any clinical trial. To minimize this risk we will collect our database only on partners-protected computers; we will not include any protected health information in our primary database or in any study documents; the key linking the patient's study ID# with their name and medical record number will be kept in a separate folder on a partners-protected computer. The key linking the treatment group with the study ID will be kept on a separate password-protected computer by the BWH research pharmacy. Biological specimen tubes containing plasma and urine will be tagged with study ID#'s only and will not include any protected health information. Specimens will be stored in locked freezers at Brigham and Women's hospital.

Storing of genetic samples

Genetic samples will be stored in locked freezers at Brigham and Women's Hospital and kept for a maximum of 25 years. The samples will be labeled with a study ID only; no protected health information will be shared with collaborators outside of Partners. Genetic data will not be placed onto publicly available repositories such as the National Institutes of Health (NIH) central repository.

D. Device complications/malfunctions

Not applicable

E. Psychosocial risks

Not applicable

F. Radiation risks

Not applicable

VIII. POTENTIAL BENEFITS

A. Potential benefits to participating individuals

Individuals receiving calcifediol or calcitriol may derive benefit through the drug's actions on immune/inflammatory pathways that are directly involved in the pathogenesis of AKI. Our hypothesis that these pharmacologic agents may prevent/attenuate AKI in critically ill patients is supported by ample data from preclinical studies and preliminary data in humans (discussed in detail in section I).

B. Potential benefits to society

Potential benefits to society include elucidation of the physiology of vitamin D metabolism in critically ill patients. Moreover, this study will help answer important questions about mechanisms responsible for the association between vitamin D deficiency and adverse outcomes, including AKI, in critically ill patients. Understanding whether the immune/inflammatory response can be enhanced by calcifediol or calcitriol will inform the research community whether a larger study of either agent, powered to evaluate hard end points such as mortality, is justified.

IX. MONITORING AND QUALITY ASSURANCE

A. Independent monitoring of source data

Data collection will be performed by study investigators in a blinded fashion. The patient assignment key linking study ID# with treatment group assignment will be maintained securely by the BWH research pharmacy throughout the data collection period.

B. Safety monitoring

We have assembled a DSMB consisting of Dr. Gearoid McMahon, a faculty member in the BWH Division of Nephrology, and Dr. Benjamin Raby, a faculty member in the BWH Division of Pulmonary and Critical Care Medicine. Drs. McMahon and Raby both have experience providing care to critically ill patients and also have expertise in patient-oriented research, clinical trials, and biostatistics. The DSMB will meet every 6 months to review unblinded data on all adverse events, with an emphasis on serum total calcium levels, and will submit a report to the IRB after each meeting. The DSMB will be responsible for determining if the research should be altered or stopped.

Additionally, the PI (Dr. David Leaf) will review daily serum creatinine values for the first 7 days in real-time for each patient enrolled, and will take appropriate action as needed for any patient who develops hypercalcemia.

C. Outcomes monitoring

Not applicable. The primary end point is a biomarker, SCr, and will not be assessed until study completion.

D. Adverse event reporting

All adverse events and unanticipated problems involving risks to subjects and others will be reported to the PHRC in accordance with PHRC adverse event and unanticipated problems reporting guidelines:

http://navigator.partners.org/ClinicalResearch/Reporting_Unanticipated_Problems_including_Adverse_Events.pdf

We will report any adverse events to the PHRC within 5 working days/7 calendar days of the date the investigator first becomes aware of the problem. Other adverse events and minor protocol deviations will be reported during continuing review.

Additionally, in accordance with IND regulations, we will report all adverse events to the FDA in an IND safety report as soon as possible, but no later than 15 calendar days after the primary investigator (David Leaf, MD) becomes aware of the safety information and determines that the information qualifies for reporting:

http://www.fda.gov/downloads/Drugs/.../Guidances/UCM227351.pdf

X. REFERENCES

1. Chertow GM, Burdick E, Honour M, Bonventre JV, Bates DW. Acute kidney injury, mortality, length of stay, and costs in hospitalized patients. J Am Soc Nephrol. 2005; 16(11):3365-70.
2. Waikar SS, Curhan GC, Wald R, McCarthy EP, Chertow GM. Declining mortality in patients with acute renal failure, 1988 to 2002. J Am Soc Nephrol. 2006; 17(4):1143-50.
3. Waikar SS, Liu KD, Chertow GM. The incidence and prognostic significance of acute kidney injury. Curr Opin Nephrol Hypertens. 2007; 16(3):227-36.
4. Masumoto O, Ohyama Y, Okuda K. Purification and characterization of vitamin D 25-hydroxylase from rat liver mitochondria. J Biol Chem. 1988; 263(28):14256-60.
5. Kawashima H, Torikai S, Kurokawa K. Localization of 25-hydroxyvitamin D3 1 alpha-hydroxylase and 24-hydroxylase along the rat nephron. Proc Natl Acad Sci U S A. 1981; 78(2):1199-203.
6. Inoue Y, Segawa H, Kaneko I, et al. Role of the vitamin D receptor in FGF23 action on phosphate metabolism. Biochem J. 2005; 390(Pt 1):325-31.
7. Wang Y, Zhu J, DeLuca HF. Where is the vitamin D receptor? Arch Biochem Biophys. 2012; 523(1):123-33.
8. Ramagopalan SV, Heger A, Berlanga AJ, et al. A ChIP-seq defined genome-wide map of vitamin D receptor binding: associations with disease and evolution. Genome Res. 2010; 20(10):1352-60.
9. Bouillon R, Lieben L, Mathieu C, Verstuyf A, Carmeliet G. Vitamin D action: lessons from VDR and Cyp27b1 null mice. Pediatr Endocrinol Rev. 2013; 10 Suppl 2:354-66.
10. Leaf DE, Waikar SS, Wolf M, et al. Dysregulated Mineral Metabolism in Patients with Acute Kidney Injury and Risk of Adverse Outcomes. Clin Endocrinol (Oxf). 2013; 79(4):491-8.
11. Arnson Y, Gringauz I, Itzhaky D, Amital H. Vitamin D deficiency is associated with poor outcomes and increased mortality in severely ill patients. QJM. 2012; 105:633-9.
12. Braun A, Chang D, Mahadevappa K, et al. Association of low serum 25-hydroxyvitamin D levels and mortality in the critically ill. Crit Care Med. 2011; 39(4):671-7.
13. Matthews LR, Ahmed Y, Wilson KL, Griggs DD, Danner OK. Worsening severity of vitamin D deficiency is associated with increased length of stay, surgical intensive care unit cost, and mortality rate in surgical intensive care unit patients. Am J Surg. 2012; 204:37-43.
14. Leaf DE, Raed A, Donnino MW, Ginde AA, Waikar SS. Randomized Controlled Trial of Calcitriol in Severe Sepsis. Am J Respir Crit Care Med. 2014; 190(5):533-41.
15. Maines MD, Mayer RD, Ewing JF, McCoubrey WK, Jr. Induction of kidney heme oxygenase-1 (HSP32) mRNA and protein by ischemia/reperfusion: possible role of heme as both promotor of tissue damage and regulator of HSP32. J Pharmacol Exp Ther. 1993; 264(1):457-62.
16. Nath KA. Heme oxygenase-1 and acute kidney injury. Curr Opin Nephrol Hypertens. 2014; 23(1):17-24.
17. Tracz MJ, Juncos JP, Grande JP, et al. Renal hemodynamic, inflammatory, and apoptotic responses to lipopolysaccharide in HO-1-/- mice. Am J Pathol. 2007; 170(6):1820-30.
18. Milwid JM, Ichimura T, Li M, et al. Secreted factors from bone marrow stromal cells upregulate IL-10 and reverse acute kidney injury. Stem Cells Int. 2012; 2012:392050.
19. Togel F, Hu Z, Weiss K, et al. Administered mesenchymal stem cells protect against ischemic acute renal failure through differentiation-independent mechanisms. Am J Physiol Renal Physiol. 2005; 289(1):F31-42.

20. Lemire JM, Archer DC, Beck L, Spiegelberg HL. Immunosuppressive actions of 1,25-dihydroxyvitamin D3: preferential inhibition of Th1 functions. J Nutr. 1995; 125(6 Suppl):1704S-8S.
21. Palmer MT, Lee YK, Maynard CL, et al. Lineage-specific effects of 1,25-dihydroxyvitamin D(3) on the development of effector CD4 T cells. J Biol Chem. 2011; 286(2):997-1004.
22. Cantorna MT. Mechanisms underlying the effect of vitamin D on the immune system. Proc Nutr Soc. 2010; 69(3):286-9.
23. van Hamburg JP, Asmawidjaja PS, Davelaar N, et al. TNF blockade requires 1,25(OH)2D3 to control human Th17-mediated synovial inflammation. Ann Rheum Dis. 2012; 71(4):606-12.
24. Griffin MD, Xing N, Kumar R. Gene expression profiles in dendritic cells conditioned by 1alpha,25-dihydroxyvitamin D3 analog. J Steroid Biochem Mol Biol. 2004; 89-90(1-5):443-8.
25. Zhang Y, Leung DY, Richers BN, et al. Vitamin D inhibits monocyte/macrophage proinflammatory cytokine production by targeting MAPK phosphatase-1. J Immunol. 2012; 188(5):2127-35.
26. Boonstra A, Barrat FJ, Crain C, et al. 1alpha,25-Dihydroxyvitamin d3 has a direct effect on naive CD4(+) T cells to enhance the development of Th2 cells. J Immunol. 2001; 167(9):4974-80.
27. Overbergh L, Decallonne B, Waer M, et al. 1alpha,25-dihydroxyvitamin D3 induces an autoantigen-specific T-helper 1/T-helper 2 immune shift in NOD mice immunized with GAD65 (p524-543). Diabetes. 2000; 49(8):1301-7.
28. van Halteren AG, van Etten E, de Jong EC, et al. Redirection of human autoreactive T-cells Upon interaction with dendritic cells modulated by TX527, an analog of 1,25 dihydroxyvitamin D(3). Diabetes. 2002; 51(7):2119-25.
29. Canning MO, Grotenhuis K, de Wit H, Ruwhof C, Drexhage HA. 1-alpha,25-Dihydroxyvitamin D3 (1,25(OH)(2)D(3)) hampers the maturation of fully active immature dendritic cells from monocytes. Eur J Endocrinol. 2001; 145(3):351-7.
30. Matilainen JM, Husso T, Toropainen S, et al. Primary effect of 1alpha,25(OH)(2)D(3) on IL-10 expression in monocytes is short-term down-regulation. Biochim Biophys Acta. 2010; 1803(11):1276-86.
31. Kovalenko PL, Zhang Z, Cui M, Clinton SK, Fleet JC. 1,25 dihydroxyvitamin D-mediated orchestration of anticancer, transcript-level effects in the immortalized, non-transformed prostate epithelial cell line, RWPE1. BMC Genomics. 2010; 11:26.
32. Jeffery LE, Burke F, Mura M, et al. 1,25-Dihydroxyvitamin D3 and IL-2 combine to inhibit T cell production of inflammatory cytokines and promote development of regulatory T cells expressing CTLA-4 and FoxP3. J Immunol. 2009; 183(9):5458-67.
33. Barrat FJ, Cua DJ, Boonstra A, et al. In vitro generation of interleukin 10-producing regulatory CD4(+) T cells is induced by immunosuppressive drugs and inhibited by T helper type 1 (Th1)- and Th2-inducing cytokines. J Exp Med. 2002; 195(5):603-16.
34. Xia JB, Wang CZ, Ma JX, An XJ. [Immunoregulatory role of 1, 25-dihydroxyvitamin D(3)-treated dendritic cells in allergic airway inflammation]. Zhonghua Yi Xue Za Zhi. 2009; 89(8):514-8.
35. Kinsey GR, Sharma R, Huang L, et al. Regulatory T cells suppress innate immunity in kidney ischemia-reperfusion injury. J Am Soc Nephrol. 2009; 20(8):1744-53.
36. Lee H, Nho D, Chung HS, et al. CD4+CD25+ regulatory T cells attenuate cisplatin-induced nephrotoxicity in mice. Kidney Int. 2010; 78(11):1100-9.
37. Cho WY, Choi HM, Lee SY, et al. The role of Tregs and CD11c(+) macrophages/dendritic cells in ischemic preconditioning of the kidney. Kidney Int. 2010; 78(10):981-92.

38. Lee JW, Kim SC, Ko YS, et al. Renoprotective effect of paricalcitol via a modulation of the TLR4-NF-kappaB pathway in ischemia/reperfusion-induced acute kidney injury. Biochem Biophys Res Commun. 2014; 444(2):121-7.
39. Azak A, Huddam B, Haberal N, et al. Effect of novel vitamin D receptor activator paricalcitol on renal ischaemia/reperfusion injury in rats. Ann R Coll Surg Engl. 2013; 95(7):489-94.
40. Sezgin G, Ozturk G, Guney S, Sinanoglu O, Tuncdemir M. Protective effect of melatonin and 1,25-dihydroxyvitamin D3 on renal ischemia-reperfusion injury in rats. Ren Fail. 2013; 35(3):374-9.
41. Park JW, Bae EH, Kim IJ, et al. Renoprotective effects of paricalcitol on gentamicin-induced kidney injury in rats. Am J Physiol Renal Physiol. 2010; 298(2):F301-13.
42. Park JW, Bae EH, Kim IJ, et al. Paricalcitol attenuates cyclosporine-induced kidney injury in rats. Kidney Int. 2010; 77(12):1076-85.
43. Park JW, Cho JW, Joo SY, et al. Paricalcitol prevents cisplatin-induced renal injury by suppressing apoptosis and proliferation. Eur J Pharmacol. 2012; 683(1-3):301-9.
44. Makibayashi K, Tatematsu M, Hirata M, et al. A vitamin D analog ameliorates glomerular injury on rat glomerulonephritis. Am J Pathol. 2001; 158(5):1733-41.
45. Tan X, Li Y, Liu Y. Paricalcitol attenuates renal interstitial fibrosis in obstructive nephropathy. J Am Soc Nephrol. 2006; 17(12):3382-93.
46. Shaffer JA, Edmondson D, Wasson LT, et al. Vitamin D supplementation for depressive symptoms: a systematic review and meta-analysis of randomized controlled trials. Psychosom Med. 2014; 76(3):190-6.
47. Bjelakovic G, Gluud LL, Nikolova D, et al. Vitamin D supplementation for prevention of mortality in adults. Cochrane Database Syst Rev. 2014(1):CD007470.
48. Zheng Y, Zhu J, Zhou M, et al. Meta-analysis of long-term vitamin D supplementation on overall mortality. PLoS One. 2013; 8(12):e82109.
49. Jiang WL, Gu HB, Zhang YF, et al. Vitamin D Supplementation in the Treatment of Chronic Heart Failure: A Meta-analysis of Randomized Controlled Trials. Clin Cardiol. 2016; 39(1):56-61.
50. Amrein K, Schnedl C, Holl A, et al. Effect of High-Dose Vitamin D3 on Hospital Length of Stay in Critically Ill Patients With Vitamin D Deficiency: The VITdAL-ICU Randomized Clinical Trial. JAMA. 2014 [Epub ahead of print].
51. Adams JS, Beeker TG, Hongo T, Clemens TL. Constitutive expression of a vitamin D 1-hydroxylase in a myelomonocytic cell line: a model for studying 1,25-dihydroxyvitamin D production in vitro. J Bone Miner Res. 1990; 5(12):1265-9.
52. Inaba M, Burgos-Trinidad M, DeLuca HF. Characteristics of the 25-hydroxyvitamin D3- and 1,25-dihydroxyvitamin D3-24-hydroxylase(s) from HL-60 cells. Arch Biochem Biophys. 1991; 284(2):257-63.
53. Smolders J, Damoiseaux J. Vitamin D as a T-cell modulator in multiple sclerosis. Vitam Horm. 2011; 86:401-28.
54. Adams JS, Hewison M. Unexpected actions of vitamin D: new perspectives on the regulation of innate and adaptive immunity. Nat Clin Pract Endocrinol Metab. 2008; 4(2):80-90.
55. Amrein K, Sourij H, Wagner G, et al. Short-term effects of high-dose oral vitamin D3 in critically ill vitamin D deficient patients: a randomized, double-blind, placebo-controlled pilot study. Crit Care. 2011; 15(2):R104.
56. Schnedl C, Dobnig H, Quraishi SA, McNally D, Amrein K. Active and native vitamin D in critical illness [letter]. Am J Respir Crit Care Med. 2014 [Epub ahead of print].
57. Stamp TC, Round JM, Haddad JG. Effect of oral vitamin D, 25-hydroxycholecalciferol (25-HCC) and whole-body ultra-violet irradiation on plasma 25-HCC levels in man. Clin Sci. 1973; 44(2):3P-4P passim.

58. Haddad JG, Jr., Rojanasathit S. Acute administration of 25-hydroxycholecalciferol in man. J Clin Endocrinol Metab. 1976; 42(2):284-90.
59. Hasegawa H, Nagano N, Urakawa I, et al. Direct evidence for a causative role of FGF23 in the abnormal renal phosphate handling and vitamin D metabolism in rats with early-stage chronic kidney disease. Kidney Int. 2010; 78(10):975-80.
60. Bacchetta J, Sea JL, Chun RF, et al. Fibroblast growth factor 23 inhibits extrarenal synthesis of 1,25-dihydroxyvitamin D in human monocytes. J Bone Miner Res. 2013; 28(1):46-55.
61. Leaf DE, Wolf M, Waikar SS, et al. FGF-23 Levels in Patients with AKI and Risk of Adverse Outcomes. Clin J Am Soc Nephrol. 2012; 7(8):1217-23.
62. Braun AB, Gibbons FK, Litonjua AA, Giovannucci E, Christopher KB. Low serum 25-hydroxyvitamin D at critical care initiation is associated with increased mortality. Crit Care Med. 2012; 40(1):63-72.
63. Prietl B, Treiber G, Mader JK, et al. High-dose cholecalciferol supplementation significantly increases peripheral CD4(+) Tregs in healthy adults without negatively affecting the frequency of other immune cells. Eur J Nutr. 2014; 53(3):751-9.
64. Kinsey GR, Sharma R, Okusa MD. Regulatory T cells in AKI. J Am Soc Nephrol. 2013; 24(11):1720-6.
65. Han JE, Jones JL, Tangpricha V, et al. High Dose Vitamin D Administration in Ventilated Intensive Care Unit Patients: A Pilot Double Blind Randomized Controlled Trial. J Clin Transl Endocrinol. 2016; 4:59-65.
66. Martinesi M, Bruni S, Stio M, Treves C. 1,25-Dihydroxyvitamin D3 inhibits tumor necrosis factor-alpha-induced adhesion molecule expression in endothelial cells. Cell Biol Int. 2006; 30(4):365-75.
67. Heikkinen S, Vaisanen S, Pehkonen P, et al. Nuclear hormone 1alpha,25-dihydroxyvitamin D3 elicits a genome-wide shift in the locations of VDR chromatin occupancy. Nucleic Acids Res. 2011; 39(21):9181-93.
68. Suzuki T, Tazoe H, Taguchi K, et al. DNA microarray analysis of changes in gene expression induced by 1,25-dihydroxyvitamin D3 in human promyelocytic leukemia HL-60 cells. Biomed Res. 2006; 27(3):99-109.
69. Xu S, Chen YH, Tan ZX, et al. Vitamin D3 pretreatment alleviates renal oxidative stress in lipopolysaccharide-induced acute kidney injury. J Steroid Biochem Mol Biol. 2015; 152:133-41.
70. Kapil A, Singh JP, Kaur T, Singh B, Singh AP. Involvement of peroxisome proliferator-activated receptor gamma in vitamin D-mediated protection against acute kidney injury in rats. J Surg Res. 2013; 185(2):774-83.
71. Mathieu C, Waer M, Laureys J, Rutgeerts O, Bouillon R. Prevention of autoimmune diabetes in NOD mice by 1,25 dihydroxyvitamin D3. Diabetologia. 1994; 37(6):552-8.
72. Branisteanu DD, Waer M, Sobis H, et al. Prevention of murine experimental allergic encephalomyelitis: cooperative effects of cyclosporine and 1 alpha, 25-(OH)2D3. J Neuroimmunol. 1995; 61(2):151-60.
73. Deluca HF, Prahl JM, Plum LA. 1,25-Dihydroxyvitamin D is not responsible for toxicity caused by vitamin D or 25-hydroxyvitamin D. Arch Biochem Biophys. 2011; 505(2):226-30.
74. Stamp TC. Intestinal absorption of 25-hydroxycholecalciferol. Lancet. 1974; 2(7873):121-3.
75. Weisman Y, Schen RJ, Eisenberg Z, et al. Single oral high-dose vitamin D3 prophylaxis in the elderly. J Am Geriatr Soc. 1986; 34(7):515-8.
76. Petkovich M, Melnick J, White J, et al. Modified-release oral calcifediol corrects vitamin D insufficiency with minimal CYP24A1 upregulation. J Steroid Biochem Mol Biol. 2015; 148:283-9.

77. Bell NH. 25-Hydroxyvitamin D3 reverses alteration of the vitamin D-endocrine system in blacks. Am J Med. 1995; 99(6):597-9.
78. Lathers DM, Clark JI, Achille NJ, Young MR. Phase 1B study to improve immune responses in head and neck cancer patients using escalating doses of 25-hydroxyvitamin D3. Cancer Immunol Immunother. 2004; 53(5):422-30.
79. Orwoll ES, McClung MR, Oviatt SK, Recker RR, Weigel RM. Histomorphometric effects of calcium or calcium plus 25-hydroxyvitamin D3 therapy in senile osteoporosis. J Bone Miner Res. 1989; 4(1):81-8.
80. Sprague SM, Crawford PW, Melnick JZ, et al. Use of Extended-Release Calcifediol to Treat Secondary Hyperparathyroidism in Stages 3 and 4 Chronic Kidney Disease. Am J Nephrol. 2016; 44(4):316-25.
81. Vieth R. Vitamin D toxicity, policy, and science. J Bone Miner Res. 2007; 22 Suppl 2:V64-8.
82. Blank S, Scanlon KS, Sinks TH, Lett S, Falk H. An outbreak of hypervitaminosis D associated with the overfortification of milk from a home-delivery dairy. Am J Public Health. 1995; 85(5):656-9.
83. Muindi JR, Peng Y, Potter DM, et al. Pharmacokinetics of high-dose oral calcitriol: results from a phase 1 trial of calcitriol and paclitaxel. Clin Pharmacol Ther. 2002; 72(6):648-59.
84. Beer TM, Myrthue A, Garzotto M, et al. Randomized study of high-dose pulse calcitriol or placebo prior to radical prostatectomy. Cancer Epidemiol Biomarkers Prev. 2004; 13(12):2225-32.
85. Beer TM, Munar M, Henner WD. A Phase I trial of pulse calcitriol in patients with refractory malignancies: pulse dosing permits substantial dose escalation. Cancer. 2001; 91(12):2431-9.
86. Fakih MG, Trump DL, Muindi JR, et al. A phase I pharmacokinetic and pharmacodynamic study of intravenous calcitriol in combination with oral gefitinib in patients with advanced solid tumors. Clin Cancer Res. 2007; 13(4):1216-23.
87. Muindi JR, Johnson CS, Trump DL, et al. A phase I and pharmacokinetics study of intravenous calcitriol in combination with oral dexamethasone and gefitinib in patients with advanced solid tumors. Cancer Chemother Pharmacol. 2009; 65(1):33-40.
88. Kidney Disease; Improving Global Outcomes (KDIGO) Acute Kidney Injury Work Group. KDIGO clinical practice guideline for acute kidney injury. Kidney Int. 2012; Suppl 2:1-138.
89. Endre ZH, Walker RJ, Pickering JW, et al. Early intervention with erythropoietin does not affect the outcome of acute kidney injury (the EARLYARF trial). Kidney Int. 2010; 77(11):1020-30.
90. Group FHNT, Chertow GM, Levin NW, et al. In-center hemodialysis six times per week versus three times per week. N Engl J Med. 2010; 363(24):2287-300.
91. Kidney Disease; Improving Global Outcomes (KDIGO) Acute Kidney Injury Work Group. KDIGO clinical practice guideline for acute kidney injury. Kidney Int Suppl 2. 2012:1-138.
92. Yadav H, Harrison AM, Hanson AC, et al. Improving the Accuracy of Cardiovascular Component of the Sequential Organ Failure Assessment Score. Crit Care Med. 2015; 43(7):1449-57.
93. Steele T, Kolamunnage-Dona R, Downey C, Toh CH, Welters I. Assessment and clinical course of hypocalcemia in critical illness. Crit Care. 2013; 17(3):R106.
